# E3 ubiquitin ligase RNF5 attenuates pathological cardiac hypertrophy through STING

**DOI:** 10.1038/s41419-022-05231-8

**Published:** 2022-10-21

**Authors:** Lu-Lu Yang, Wen-Chang Xiao, Huan Li, Zheng-Yang Hao, Gui-Zhi Liu, Dian-Hong Zhang, Lei-Ming Wu, Zheng Wang, Yan-Qing Zhang, Zhen Huang, Yan-Zhou Zhang

**Affiliations:** 1 Cardiovascular Hospital, the First Affiliated Hospital of Zhengzhou University, Zhengzhou University, Zhengzhou, 450052 China; 2 Department of Cardiovascular Surgery, Huanggang Central Hospital, Huanggang Institute of Translational Medicine, Huanggang, 438000 China

**Keywords:** Heart failure, Ubiquitylation

## Abstract

Ring-finger protein 5 (RNF5) is an E3 ubiquitin ligase which is expressed in a variety of human tissues. RNF5 is involved in the regulation of endoplasmic reticulum stress, inflammation, and innate immunity and plays an important role in the occurrence and development of various tumors. However, the role of RNF5 in cardiac hypertrophy has not been reported. In this study, we found the expression of RNF5 was increased in the hearts of mice with pathological cardiac hypertrophy. The loss-of-function research demonstrated that RNF5 deficiency exacerbated cardiac hypertrophy, whereas gain-of-function studies revealed that overexpression of RNF5 had opposite effects. The stimulator of interferon genes (STING) is a signaling molecule that can activate type I interferon immunity, which can meditate inflammation and immune response in many diseases. The protein–protein interaction experiments confirmed that STING interacted with RNF5. Further studies showed that RNF5 inhibited cardiac hypertrophy by promoting STING degradation through K48-linked polyubiquitination. Therefore, we defined RNF5 as importantly regulated signaling for cardiac hypertrophy.

## Introduction

Pathological cardiac hypertrophy is caused by hypertension, valvular heart disease, coronary artery disease, and hereditary cardiomyopathy [1]. Pathological cardiac hypertrophy usually progresses to heart failure and is a major risk factor for arrhythmias [2]. In high-income North America, eastern Sub-Saharan Africa, East Asia, and Southeast Asia, heart failure caused the most significant reduction in healthy life year (HeaLY) for males [3]. Despite the increased use of techniques such as open heart transplantation and mechanical circulatory support device placement, morbidity and mortality in patients with heart failure remained high [3]. Therefore, exploring the underlying mechanisms of pathological cardiac hypertrophy is crucial to delay or even reverse the progression of heart failure.

Ring-finger protein 5 (RNF5, also known as RMA1) is an E3 ubiquitin ligase, mainly located at the endoplasmic reticulum and mitochondria [4, 5]. RNF5 is anchored to the endoplasmic reticulum by its C-terminal and contains a classical RING domain (giving ligase activity) [6]. RNF5 plays an important role in endoplasmic reticulum stress response and unfolded protein response [7]. Cystic fibrosis is associated with the misfolding and premature degradation of the cystic fibrosis transmembrane conductance regulator (CFTR) mutant CFTRΔF508, it has been demonstrated that RNF5 targeted CFTRΔF508 for degradation through the endoplasmic reticulum-associated degradation (ERAD) [8, 9]. Virus-induced signaling adapter (VISA, also known as MAVS) is a critical adapter protein to RNA virus and plays a significant role in the innate immune responses of the host [10]. RNF5 modulates the cellular antiviral responses by K48-linked polyubiquitination and degradation of VISA [5, 11]. A recent study has shown that RNF5 ameliorated nonalcoholic steatohepatitis (NASH) through ubiquitin-mediated degradation of 3-hydroxy-3-methylglutaryl CoA reductase degradation protein 1 [ref. 12]. RNF5 is closely associated with proliferation, apoptosis, and autophagy. However, current studies on RNF5 have mainly focused on tumor and innate immune responses, and the role of RNF5 in pathological cardiac hypertrophy remains largely unknown.

In this study, we find that RNF5 expression is upregulated in animal and cellular models of cardiac hypertrophy. After pathological stimulation, RNF5 deficiency significantly aggravates pathological cardiac hypertrophy, inflammatory response, and fibrosis, while overexpression of RNF5 has the opposite effects. However, RNF5 has no such effects under physiological conditions. We found a direct interaction between RNF5 and STING. STING aggravated pathological cardiac hypertrophy after PE treatment, while RNF5 could promote STING degradation through K48-linked polyubiquitination, thus alleviating cardiac hypertrophy. In conclusion, our results suggest that RNF5 may act as a promising therapeutic target in pathological cardiac hypertrophy.

## Materials and methods

### Construction of animal models

All animal use protocols were approved by Zhengzhou University. The procedures were in accordance with the National Institutes of Health Guidelines for the Care and Use of Laboratory Animals.

In order to obtain RNF5 knockout mice, we used the CRISPR online design tool (http://chopchop.cbu.uib.no/) to predict target DNA regions boot sequence-guideRNA target site: GCCCCGCTCGCGATTTGGCCCTTCGGG, RNF5-sgRNA expression vector was constructed using pUC57-sgRNA (Addgene,51132) as skeleton vector. The in vitro transcripts of Cas9 expression vector pST1374-Cas9 (Addgene 44758) and sgRNA expression vector were purified, recovered, and configured into a mixed system (Cas9 mRNA: 10 ng/ul;sgRNA: 10 ng/ul), the mixture was injected into single-cell fertilized eggs of C57BL/6 mice by FemtoJet 5247 microinjection system, and the injected fertilized eggs were transplanted into surrogate female mice, and F0 generation mice were obtained after about 19–21 days of gestation. The ear tissues of mice 2 weeks after birth were collected, genomic DNA was extracted, and the following primers were used to identify the genotypes of mice: RNF5-Check F1: 5′ -CTGGGGGTACTGAGGGCTAC-3′, RNF5-check R1:5′-GCCCTCTGGTCATCTGAAAA-3′. The selected Founder was then multiplied and constructed until RNF5-/- mice were obtained for subsequent experiments.

### Animal surgery

Male mice aged between 9 and 11 weeks with a body weight of 25.5–27 g were randomly divided into the TAC group and sham group. Mice in the TAC group were anesthetized by intraperitoneal injection with tribromoethanol (400 mg/Kg). After no obvious toe reaction and stable and even breathing rules were observed, the mice were placed in a supine position and fixed on a self-adjusting heating pad at 37 °C. Take the junction of the clavicle and thoracic vertebra as the center, cut it and use forceps to penetrate into the incision to tear the muscles on both sides, separate the thymus on both sides and expose the aortic arch. The 7-0 silk thread was passed through the aortic arch, and the 26-G cushion needle was placed parallel to the top of the aortic arch. After ligation of the blood vessels and the cushion needle, the cushion needle was pulled out to form a narrowing of the aortic arch. Animals in the sham group underwent all steps except aortic ligation. After surgery, the skin at the opening was sutured, and the mice were placed in a 37 °C temperature box to wake up.

### Echocardiographic assessment

Mice were anesthetized by inhalation of isoflurane (1.5–2%) and then fixed in a supine position on a thermostatic plate. Ultrasound detection was performed using Small Animal Ultrasound Imaging System (VEVO2100, FUJIFILM VISUALSONICS, Canada) with a 30-MHz(MS400) probe. The left ventricular volume and the thickness of the left ventricular wall were measured at the papillary muscle for three consecutive cycles in M-mode echocardiography mode. The left ventricular end-systolic diameter (LVESd), left ventricular end-diastolic diameter (LVEDd), left ventricular ejection fraction (EF%), and short axis shortening rate (FS%) were measured. Echocardiography was conducted by investigators blinded to the study.

### Histomorphological staining analysis

Four weeks after TAC surgery, the mice were weighed and recorded. After the heart was removed, it was quickly placed in 10% KCl solution to stop the heart in the diastolic phase, weighed, and fixed in liquid nitrogen or 10% formalin. Lung weight and tibial length were also measured.

The mice heart was fixed for 48 h, and the cross-cut wax block was sequentially sectioned. The sections were 5 μm thick and stained with hematoxylin (G1004, Servicebio) & eosin (BA-4024, Baso) (H&E) and picrosirius red (26357-02, Hedebiotechnology) (PSR) to measure the cross-sectional area and collagen fiber content of cardiomyocytes. Image-pro Plus 6.0 software was used for measurement.

### Western blot

Left ventricular tissue or cell samples were collected and lysed by RIPA buffer (720 μL of RIPA buffer, 20 μL of phenylmethylsulfonyl fluoride, 100 μL of complete protease inhibitor cocktail, 100 μL of Phos-stop, 50 μL of NaF, and 10 μL of Na3 VO4 in a final volume of 1 mL). After lysis and centrifugation, the supernatant was taken as total protein and quantified by a BCA protein kit (Pierce). After separation by using SDS polyacrylamide gel electrophoresis, the proteins were transferred to a 0.45 μm PVDF (IPVH00010, Millipore) membrane. The membrane was then blocked with 5% nonfat milk at room temperature for 1 h. The PVDF membrane was cleaned three times with TBST, 5 min each time. A primary antibody was added and incubated at 4 °C overnight. The secondary antibodies were added the next day. ECL luminescent substrate (1705062, Bio-Rad) was used for imaging and the Bole gel imaging system (ChemiDoc XRS+) was used for signal collection. Image Lab (Version5.1) software was used to analyze the results, and the corresponding antibody information is Supplementary Table 1.

### Quantitative real-time (RT)-PCR

TRIzol reagent (15596-026, Invitrogen) was used to extract total RNA from tissue or cell samples. The RNA was reversed into cDNA using the Transcriptor First Strand cDNA Synthesis Kit (04896866001, Roche). SYBR Green PCR Master Mix (04887352001, Roche) was added and the expression of selected genes was detected by RT-PCR (Roche). GAPDH (glyceraldehyde-3-phosphate dehydrogenase) was used as the reference gene, and the primer sequences used were Supplementary Table 2.

### Construction of retrovirus vector

STING genes were subcloned into replication-deficient adenovirus vectors controlled by cytomegalovirus (CMV) promoter and used for overexpression of STING, with GFP expression as control. Replication-deficient adenovirus vectors carrying short hairpin RNA targeting RNF5 were used to knock down the expression of RNF5, while AdshRNA adenovirus was used as a control, and the adenovirus overexpressing RNF5 was purchased from Han Heng Biotechnology Co., LTD. Adenoviruses infected cardiomyocytes with a 50-particle/cell multiplicity of infection (MOI) for 24 h and were subsequently identified. Virus primer information was Supplementary Table 3.

### Cardiomyocyte culture

The hearts of 1- to 2-day-old SD rats were taken and the blood vessels were removed. The tissue blocks were cut into 1–2 cubic millimeters and digested with 0.125% trypsin to obtain neonatal rat cardiomyocytes (NRCMs). Added DMEM/F12 (C11330, Gibco) medium (10% fetal bovine serum (FBS, 10099141C, GIBCO), 1% penicillin/streptomycin (15140-122, GIBCO), and 5-bromodeoxyuridine (0.1 mM, to inhibit fibroblast proliferation, B5002-250MG, sigma)) for 24 h. NRCMs infected with adenovirus were treated with serum-free medium for 12 h, and then stimulated with 50 μM PE (PHR1017, Sigma) for 24 h. The control group was added with the same amount of PBS. The whole cell culture process was carried out at 37.0 °C and 5% CO_2_.

### Immunofluorescence staining

NRCMs were cultured for 24 h and fixed with 4% formaldehyde (G1101-500ML, Servicebio) for 30 min, then permeated with 0.2%Triton X-100 in PBS and blocked with 10% BSA (BAH66-0100, Equitech Bio) at 37.0 °C. The cells on slides were incubated with α-actinin antibody (05-384, Merck Millipore, 1:100 dilution), followed by staining with a fluorescent secondary antibody (donkey anti-mouse IgG [H + L] secondary antibody, A21202, Invitrogen, 1:200) and then the slides were mounted with an antifade mounting medium containing DAPI. Cell surface area was measured using image-Pro Plus 6.0 software.

### Transcriptome analysis

For RNA-SEQ sequencing, the total RNA of the sample was first extracted and the cDNA library was constructed. MGISEQ-2000 RS was used for RNA-sequencing of the single-end library, and the reading length was 50 bp. HISAT2 software (Version 2.1.0) was used to compare the sequence fragments to the mouse reference genome (mm10/GRCm38). The resulting files were then transformed by SAMtools (Version 1.4) into a binary BAM format that can store the comparison information. Next, the exon model value per kilobase fragment per million gene location fragment (FPKM) for each identified gene is calculated using the default parameter of StringTie (Version 1.3.3b). Then, DESeq2 (version 1.2.10) identified differentially expressed genes (DEG) based on the following two criteria:(1) multiple changes greater than 1.5; (2) The corresponding corrected *p* < 0.05.

### Hierarchical clustering analysis

In hierarchical clustering analysis, the similarity between different samples was calculated, and the Unweighted Pair Group Method With Arithmetic Mean (UPGMA) algorithm was used to establish the hierarchical nested clustering tree, and then the hclust function of the R package was used for visualization.

### Gene set enrichment analysis

Gene set enrichment analysis (GSEA) uses the gene set in the KEGG pathway to sequence genes according to the degree of differential expression. Then check whether the gene sets are concentrated at the top or bottom of the sequencing list to obtain the overall expression changes of these gene sets. The analysis was performed on the Java GSEA (Version 3.0) platform using the “Signal2Noise” metric, and gene sets with *p* < 0.05 and FDR <0.25 were considered statistically significant.

### Co-IP

First, the required plasmids were co-transfected in 293 T or indicated adenovirus were infected with NRCMs, and the primer information of plasmids was Supplementary Table 4. Twenty-four hours after plasmid transfection, IP lysis buffer (20 mM Tris-HCl, pH 7.4;150 mM NaCl; 1 mM EDTA; and 1% NP-40) were used to lysate cells. After high-speed centrifugation at 4 °C, the supernatant containing protein was incubated overnight with Protein G-agarose beads and anti-label antibodies at 4 °C. Centrifugation at 3000 rpm at 4 °C, wash beads with 300 mM and 150 mM NaCl buffers for about three times respectively, then re-suspend beads with 2x SDS loading buffer and boil them at 95 °C for 5–10 min. Then the analysis results were detected by WB.

### Ubiquitination

293 T cells co-transfected with indicated plasmids were lysed in 80 μl 150 mM IP lysis buffer and 10 μl 10% SDS lysis buffer and then denatured by heating at 95 °C for 10 min. After heating, 900 μl 150 mM IP lysis buffer were added to the lysates. And then, after sonication and centrifugation (12,000 rpm for 15 min), collected the supernatant and incubated with indicated antibody and protein G-agarose beads for 3 h at 4 °C. Washing the beads with 500 mM IP lysis buffer (20 mM Tris-HCl, pH 7.4; 500 mM NaCl; 1 mM EDTA; and 1% NP-40) for three times, after centrifugation (3000 rpm for 2 min), the beads were boiled at 95 °C with 2x SDS loading buffer for 10 min and separated on the SDS-PAGE for western blotting as previously described before.

### GST-pulldown

Flag-RNF5, GST-HA-STING, Flag-STING, and GST-HA-RNF5 were overexpressed in eukaryotic cells. The lysis solution (50 mM Na2HPO4, pH 8.0;300 mM NaCl;1% Triton X-100; Cocktail) were used to lyse cells. GST Beads were used to purify protein samples. GST-HA-STING and Flag-RNF5, GST-HA-RNF5 and Flag-STING were mixed and incubated overnight at 4 °C.Buffer solutions (20 mM; 150 mM NaCl; 0.2% Triton X-100) were used to wash beads three times, then the beads were re-suspend with 2x SDS loading buffer and boiled them at 95 °C for 5–10 min. Then the analysis results were detected by WB.

### Statistical analysis

All data in this study were statistically analyzed in the form of mean ± SD. For data that showed a normal distribution, differences between two groups were compared with a two-tailed Student’s *t*-test, a One-way analysis of variance (ANOVA) was performed for data comparison between multiple groups, and the Bonferroni test (equal variances assumed) or Tamhane’s T2 test (equal variances not assumed) was used for correction. SPSS (Statistical Package for the Social Sciences) 25.0 software was used to analyze data, and *p* < 0.05 was considered to be statistically significant.

## Results

### RNF5 expression is upregulated in cardiac hypertrophy

**Fig. 1:**
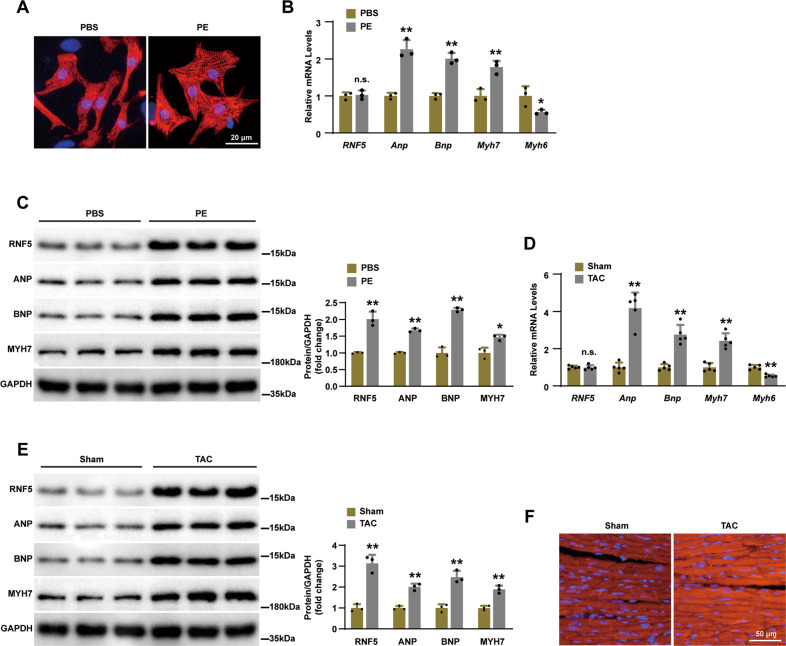
Expression of RNF5 increased in cardiac hypertrophy models. **A** The surface area of NRCMs increased after 24 h of PE treatment. Scale bars: 20 μm. **B** RT-PCR analysis of RNF5, ANP, BNP, MYH7, and MYH6 levels in NRCMs after 24 h of PE or PBS treatment. **C** WB analysis of RNF5, ANP, BNP, and MYH7 levels in NRCMs after 24 h of PE or PBS treatment. **D** RT-PCR analysis of the mRNA levels of RNF5, ANP, BNP, MYH7, and MYH6 in mice hearts after 4 weeks of TAC or sham surgery (*n* = 5 mice per group). **E** WB analysis of RNF5, ANP, BNP, and MYH7 levels in mice hearts after 4 weeks of TAC or sham surgery. **F** Immunofluorescence showed the expression of RNF5 in mice hearts after TAC or sham surgery. Scale bars: 50 μm. For (**B**–**E**) ^n.s.^*P* > 0.05 versus PBS or sham, ^*^P < 0.05 or ^**^P < 0.01 versus PBS or sham. Data were presented as the mean ± SD. Statistical analysis was carried out by Student’s two-tailed *t*-test.

To explore the role of RNF5 in the development of cardiac hypertrophy and heart failure, we treated neonatal rat cardiomyocytes (NRCMs) with phenylephrine (PE) to induce cardiomyocyte hypertrophy in vitro. Compared with phosphate buffer saline (PBS), PE stimulation significantly increased the surface area of cardiomyocytes (Fig. 1A). RT-PCR and WB showed the mRNA and protein expression of atrial natriuretic peptide (ANP), brain natriuretic peptide (BNP) and myosin heavy chain 7 (MYH7) were upregulated and the mRNA levels of myosin heavy chain 6 (MYH6) were downregulated in PE treated samples compared with those treated with PBS (Fig. 1B, C). With regard to RNF5, although the expression level of mRNA was not significantly different, the expression of the protein was significantly increased (Fig. 1B, C). On the other hand, cardiac tissue testing of these indicators yielded similar results after transverse aortic coarctation (TAC) surgery in wild-type (WT) mice (Fig. 1D, E). Further, immunofluorescence tests showed that the expression of RNF5 was significantly upregulated in heart sections of WT mice treated with TAC surgery (Fig. 1F). Altogether, the increased expression of RNF5 in cardiac hypertrophy samples suggests that RNF5 may be involved in the pathogenesis of cardiac hypertrophy.

### RNF5 deficiency promotes cardiac remodeling

**Fig. 2:**
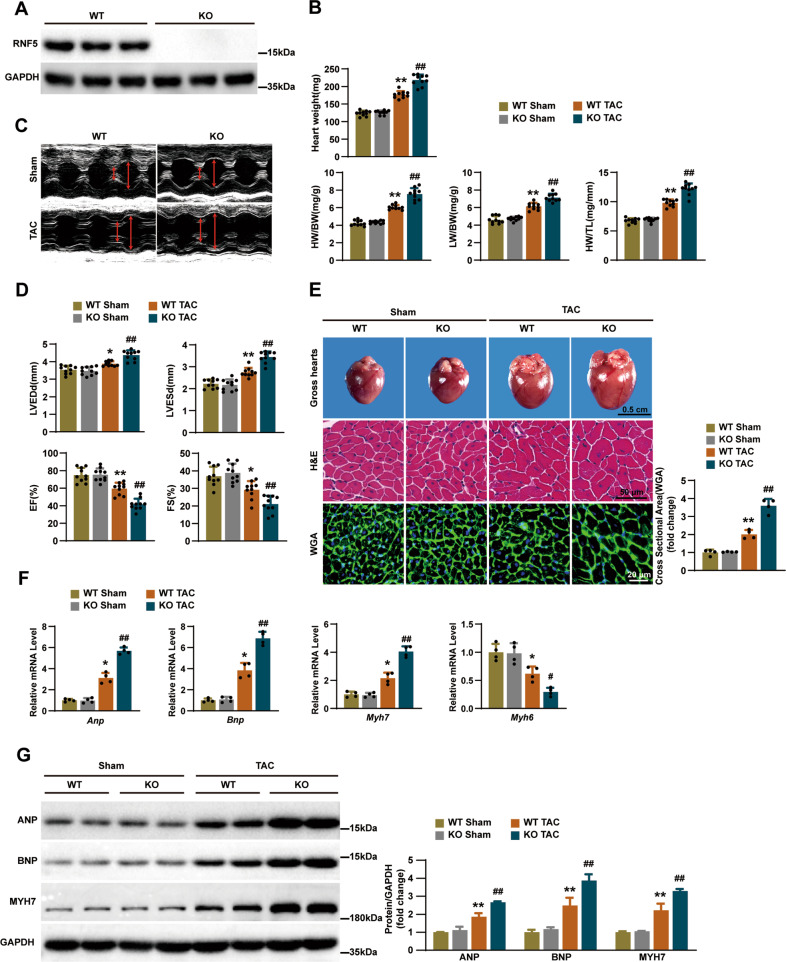
RNF5 deficiency aggravates TAC-induced cardiac remodeling. **A** WB showed the expression of RNF5 in the hearts of RNF5 KO and WT mice. **B** Statistical results of the Heart weight, HW/BW, LW/BW, and HW/TL ratios of the indicated groups (*n* = 10 mice per group). **C**, **D** Echocardiography measurements and representative images in the indicated groups (*n* = 10 mice per group). **E** Gross hearts, HE, and WGA staining in WT and RNF5 KO mice 4 weeks after TAC or sham surgery (*n* = 6, 6, 4 mice per group). Scale bars: 0.5 cm, 50 μm, 20 μm. The statistical results of cardiomyocyte’s cross-sectional area were analyzed after WGA staining (*n* = 120 cells per group). **F** RT-PCR analysis of the mRNA levels of ANP, BNP, MYH7, and MYH6 in WT and RNF5 KO mice 4 weeks after TAC or sham surgery (*n* = 4 mice per group). **G** WB analysis of ANP, BNP, and MYH7 protein levels in WT and RNF5 KO mice 4 weeks after TAC or sham surgery. For (**B**–**G**) ^*^P < 0.05 or ^**^P < 0.01 versus WT sham, ^#^*P* < 0.05 or ^##^*P* < 0.01 versus WT TAC. Data were presented as the mean ± SD. Statistical analysis was carried out by one-way ANOVA.

To investigate whether RNF5 is involved in the regulation of cardiac hypertrophy, we constructed RNF5 gene knockout (KO) mice for the loss-of-function experiments (Fig. 2A). Under basal conditions, RNF5 deficiency had no significant effect on heart weight (HW), HW/body weight (BW), lung weight (LW) /BW and HW/tibia length (TL) compared with WT mice (Fig. 2B). However, after treated with TAC surgery for 4 weeks, the results showed that HW, HW/BW, LW/BW and HW/TL were significantly increased in RNF5 KO mice (Fig. 2B). Further echocardiographic examination revealed that RNF5 deficiency aggravated cardiac dysfunction in mice (Fig. 2C). Compared with WT mice, cardiac dysfunction was deteriorated in RNF5 KO mice, which was manifested by the significant increase of left ventricular end-diastolic dimension (LVEDd), left ventricular end-systolic dimension (LVESd), as well as the decrease of ejection fractions (EF) and fraction shortening (FS) (Fig. 2D). In conclusion, echocardiography and hemodynamic measurements showed that RNF5 deficiency significantly exacerbated TAC-induced cardiac dilation and dysfunction.

We next assessed cardiac pathological sections after TAC surgery. Histological examination revealed that gross hearts and the cross-sectional area of cardiomyocytes of RNF5 KO mice were increased compared with WT mice after TAC surgery (Fig. 2E). Correspondingly, compared with WT mice, mRNA and protein levels of hypertrophic marker genes (ANP, BNP, and MYH7) in the heart tissues of RNF5 KO mice were significantly increased after TAC surgery, and the mRNA level of MYH6 was decreased (Fig. 2F, G). These data provide further evidence that the deletion of RNF5 promotes cardiac remodeling upon pressure overload.

### Deficiency in RNF5 exacerbated TAC-induced fibrosis and inflammation

**Fig. 3:**
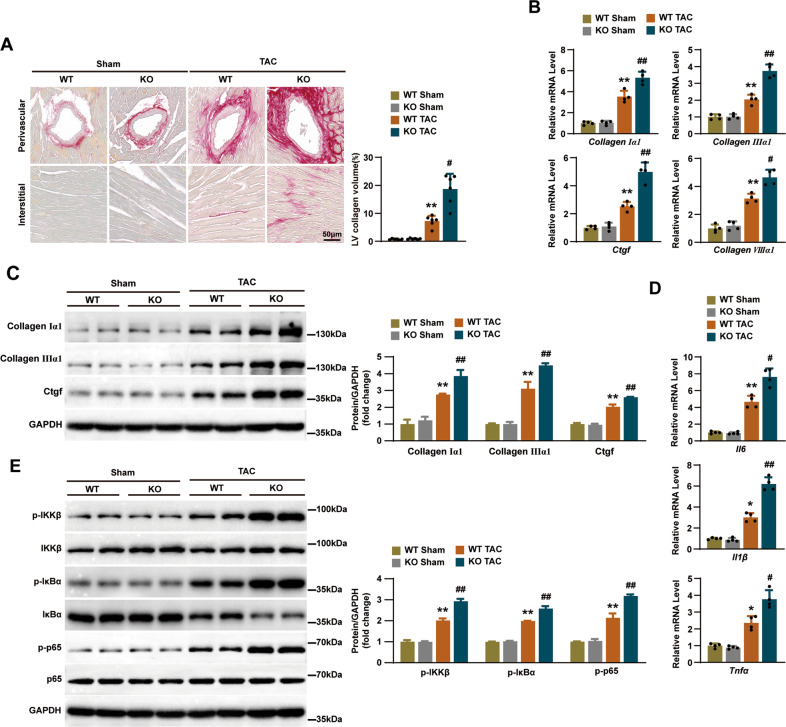
RNF5 deficiency aggravates TAC-induced myocardial fibrosis and inflammation. **A** PSR staining in WT and RNF5 KO mice 4 weeks after TAC or sham surgery and the degree of interstitial fibrosis was measured (*n* = 6 mice per group). Scale bars: 50 μm. Statistical results for the collagen volume in left ventricular interstitial tissues in different groups (*n* = 6 mice per group). **B** RT-PCR analysis of the mRNA levels of Collagen Iα1, Collagen IIIα1, CTGF, and Collagen VIIIα1 in WT and RNF5 KO mice 4 weeks after TAC or sham surgery (*n* = 4 mice per group). **C** WB analysis of Collagen Iα1, Collagen IIIα1, and CTGF protein levels in WT and RNF5 KO mice 4 weeks after TAC or sham surgery. **D** RT-PCR analysis of the mRNA levels of IL-6, IL-1β, and TNF-α in WT and RNF5 KO mice 4 weeks after TAC or sham surgery (*n* = 4 mice per group). **E** Phosphorylation and total protein expression levels of IKKβ, IkBα, and p65 in WT and RNF5 KO mice 4 weeks after TAC or sham surgery. For (**A**–**D**) ^*^P < 0.05 or ^**^P < 0.01 versus WT sham, ^#^*P* < 0.05 or ^##^*P* < 0.01 versus WT TAC. Data were presented as the mean ± SD. Statistical analysis was carried out by one-way ANOVA.

Perivascular and interstitial fibrosis are important characteristics of cardiac hypertrophy caused by pressure overload [13]. We, therefore, evaluated TAC-induced cardiac fibrosis by staining with picrosirius red (PSR) to determine the degree of fibrosis. Both interstitial and perivascular fibrosis were markedly increased in TAC-treated WT mice hearts but to a more prominent extent in RNF5 KO mice hearts after TAC surgery (Fig. 3A). Correspondingly, fibrosis was further quantified by measuring the mRNA levels of fibrosis markers (Collagen Iα1, Collagen IIIα1, connective tissue growth factor (CTGF) and Collagen VIIIα1) and the expression of protein levels of fibrosis markers (Collagen Iα1, Collagen IIIα1, and CTGF), and it was found that TAC-induced fibrosis was significantly aggravated in RNF5 KO mice than WT mice (Fig. 3B, C).

In the development of cardiac hypertrophy, the increased expression of IL-6, TNF-α, and IL-1β is closely related to Collagen I and Collagen III deposition [14]. As shown in Fig. 3D, the mRNA levels of inflammatory cytokines IL-6, IL-1β, and TNF-α were significantly higher in RNF5 KO mice than that in WT mice after TAC surgery. Activation of inflammatory signaling pathways promotes myocardial hypertrophy and fibrosis [15]. We further investigated the effects of RNF5 deficiency on NF‐κB signaling pathways. WB results showed that the phosphorylation of IKKβ, IkBα, and p65 proteins were significantly increased in RNF5 KO mice and WT mice after TAC surgery compared with the sham surgery group, and the expression of these proteins was higher in the RNF5 KO mice compared with the WT mice (Fig. 3E). These results indicate that RNF5 deficiency activates NF‐κB pathway dependent inflammatory responses under pressure overload in vivo.

### Deficiency in RNF5 promotes cardiomyocytes hypertrophy in vitro

**Fig. 4:**
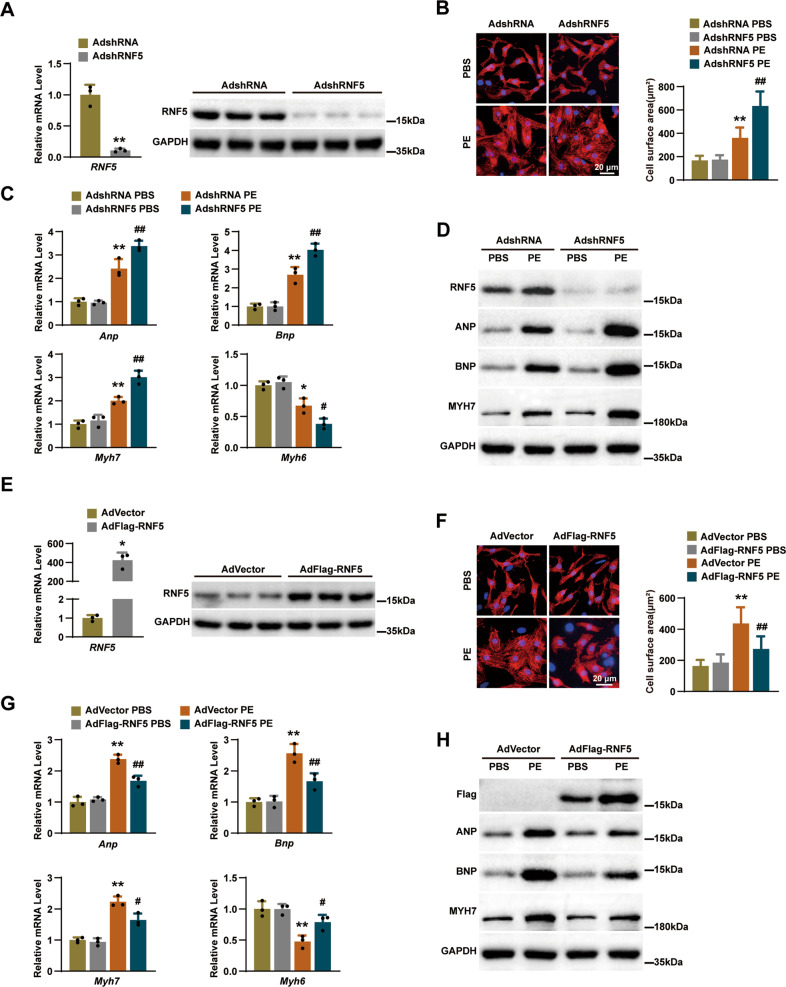
RNF5 deficiency aggravates cardiomyocyte hypertrophy in vitro. **A** NRCMs were infected with AdshRNA or AdshRNF5 for 24 h, and the expression of RNF5 in cell lysates was analyzed by RT-PCR and WB. **B** NRCMs were infected with AdshRNA or AdshRNF5, and then NRCMs were treated with PBS or PE for 24 h. Cardiomyocytes were identified by α-actinin staining (red), and nuclei were stained with DAPI (blue). Scale bars: 20 μm. Statistical results for the cell surface area of cardiomyocytes in different groups (*n* = 40 cells per group). **C** RT-PCR analysis of the mRNA levels of ANP, BNP, MYH7, and MYH6 in NRCMs infected with AdshRNA or AdshRNF5 after 24 h treatment with PBS or PE. **D** WB analysis of RNF5, ANP, BNP, and MYH7 in NRCMs infected with AdshRNA or AdshRNF5 after 24 h treatment with PBS or PE. **E–H** NRCMs were infected with AdVector or AdFlag-RNF5 for 24 h; other experimental methods and conditions were described in **A**–**D**. For (**A**, **E**) ^*^P < 0.05 versus AdVector, ^**^P < 0.01 versus AdshRNA. Data were presented as the mean ± SD. Statistical analysis was carried out by Student’s two-tailed *t*-test. For (**B**, **C**) ^*^P < 0.05 or ^**^P < 0.01 versus AdshRNA PBS, ^#^*P* < 0.05 or ^##^*P* < 0.01 versus AdshRNA PE. Data were presented as the mean ± SD. Statistical analysis was carried out by one-way ANOVA. For (**F**, **G**) ^*^P < 0.05 or ^**^P < 0.01 versus AdVector PBS, ^#^*P* < 0.05 or ^##^*P* < 0.01 versus AdVector PE. Data were presented as the mean ± SD. Statistical analysis was carried out by one-way ANOVA.

Because cardiomyocyte enlargement is the defining characteristic of cardiac remodeling, we further evaluated the specific role of RNF5 in cardiomyocytes by infecting NRCMs with adenovirus harboring RNF5 short hairpin RNA (AdshRNF5) (Fig. 4A). After 24 h of PE or PBS treatment, the surface area of cardiomyocytes was determined by α-actinin immunofluorescence staining. Compared with the control group, AdshRNF5 significantly increased PE-induced cardiomyocyte enlargement (Fig. 4B), accompanied by significantly increased expression of ANP, BNP, and MYH7 and decreased expression of MYH6 (Fig. 4C, D). Furthermore, we infected NRCMs with adenovirus overexpression with RNF5 (AdFlag-RNF5) (Fig. 4E). In contrast, overexpression of RNF5 significantly reduced hypertrophy of cardiomyocytes (Fig. 4F). Compared with the control group, overexpression of RNF5 downregulated the protein and mRNA expression of ANP, BNP, and MYH7, and upregulated the mRNA expression of MYH6 (Fig. 4G, H). These results suggest that RNF5 could relieve the hypertrophic growth of PE-induced primary cardiomyocytes.

### Role of RNF5 in the pathogenesis of cardiac hypertrophy at the transcriptomic level

**Fig. 5:**
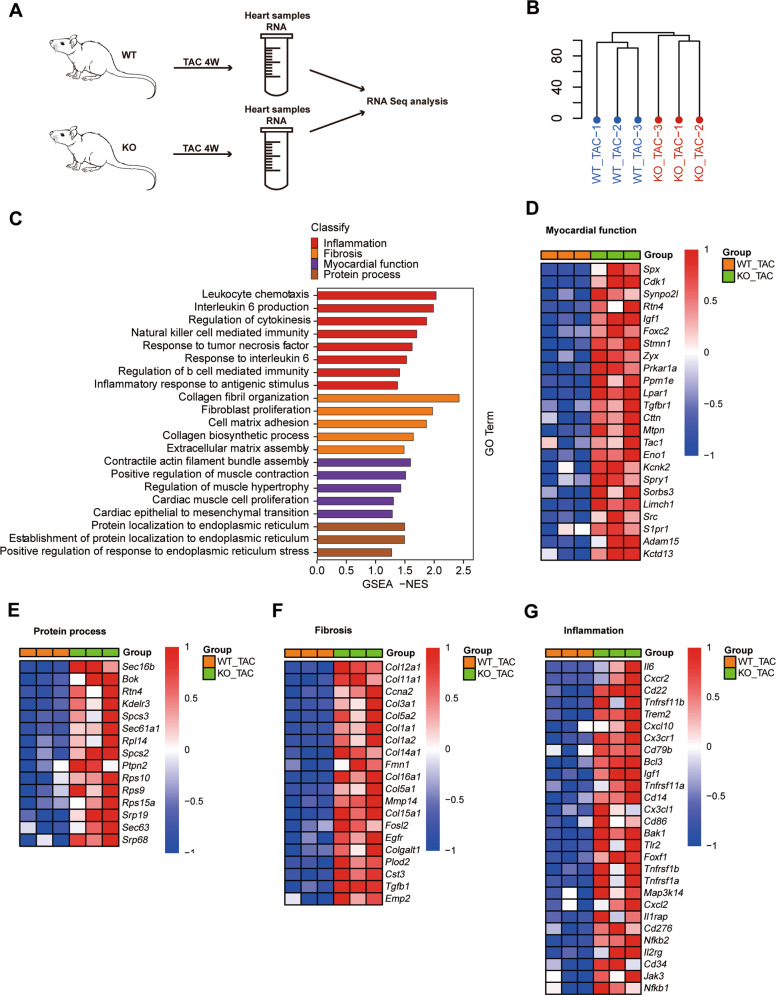
Transcriptome analysis of the mechanism of RNF5 in cardiac hypertrophy. **A** RNA-Seq construction plan for heart tissues from RNF5 KO and WT mice 4 weeks after TAC surgery. **B** Hierarchical Clustering Dendrogram of the distribution of RNA-Seq. **C** GSEA analysis of RNA-Seq data showed the pathways activated after RNF5 deficiency. **D–G** Heat maps of transcriptome analysis showed that myocardial function, protein process, fibrosis, and inflammation-related genes were upregulated by RNF5 deficiency.

To explore the role of RNF5 in the pathogenesis of pathological cardiac hypertrophy at the transcriptomic level, RNA was extracted from the heart tissues of RNF5 KO mice and WT mice after TAC surgery for RNA-sequencing (RNA-Seq) analysis (Fig. 5A). The distribution profiles of RNA-Seq was analyzed by hierarchical clustering dendrogram analysis, which showed that the samples were divided into two clusters (Fig. 5B). Gene Set Enrichment Analysis (GSEA) analysis showed that inflammation, fibrosis, myocardial function, and protein process related pathways were all activated by RNF5 deficiency (Fig. 5C). Heat maps of transcriptome analysis showed increased effective activation of genes related to myocardial function, protein process, fibrosis, and inflammation in the RNF5 KO group (Fig. 5D–G). Taken together, these results suggest that RNF5 deficiency could activate signaling pathways and genes associated with cardiac hypertrophy.

### Identification of STING as an RNF5 interacting protein

**Fig. 6:**
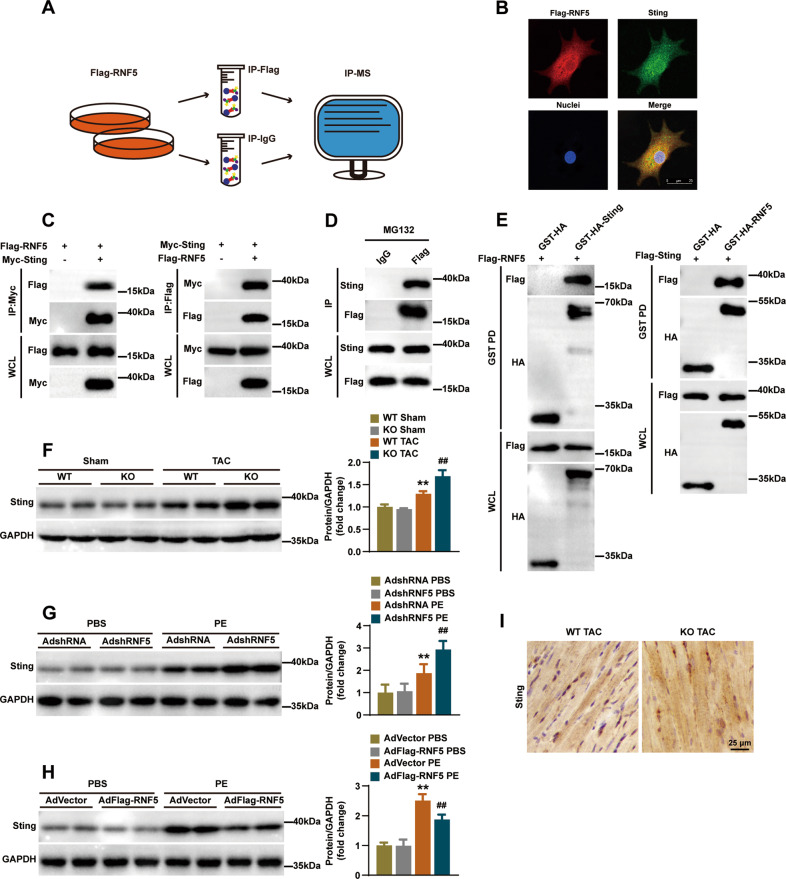
The interaction between STING and RNF5 was confirmed. **A** The flow chart shows the Co-IP and subsequent IP-MS of RNF5, screening for candidate proteins that interact with RNF5. **B** NRCMs was infected with AdFlag-RNF5 after MG132 was added 6 h before harvest, and immunofluorescence showed that Flag-RNF5 (red) co-located with STING (green). **C** After HEK-293T cells were transfected with Flag-RNF5 and Myc-STING plasmids, Co-IP detection suggested that RNF5 and STING interacted. **D** After NRCMs were infected with AdFlag-RNF5 and IgG antibody was used as a negative control, MG132 was added 6 h before harvest. Co-IP demonstrated the interaction between exogenous RNF5 and endogenous STING. **E** Glutathione S-transferase precipitation assay showed that RNF5 is directly bound to STING. **F** WB analysis of STING protein levels in WT and RNF5 KO mice 4 weeks after TAC or sham surgery. **G** WB analysis of STING in NRCMs infected with AdshRNA or AdshRNF5 after 24 h treatment with PBS or PE. **H** WB analysis of STING in NRCMs infected with AdVector or AdFlag-RNF5 after 24 h treatment with PBS or PE. **I** Immunohistochemical showed the expression of STING in the indicated groups. Scale bars: 50 μm. For (**F**–**H**) ^**^P < 0.01 versus WT Sham or AdshRNA PBS or AdVector PBS, ^##^*P* < 0.01 versus WT TAC or AdshRNA PE or AdVector PE. Data were presented as the mean ± SD. Statistical analysis was carried out by one-way ANOVA.

To explore the potential mechanism of RNF5 regulating cardiac hypertrophy, we used IP-MS to screen out candidate proteins that might bind to RNF5 (Fig. 6A). According to IP-MS, we found that stimulator of interferon genes (STING) might be associated with RNF5. During the development of pathological cardiac hypertrophy, some inflammatory signaling pathways are activated, such as NF-κB [13]. STING, also known as Transmembrane protein 173 (TMEM173), has been identified as a key molecule in antiviral responses. RNF5 has been reported to inhibit virus-triggered IRF3, NF-κB activation, and cellular antiviral responses by regulating STING [16]. Therefore, we further explored the interaction between STING and RNF5 in cardiomyocytes. Immunofluorescence staining of NRCMs showed that RNF5 and STING were primarily co-located in the cytoplasm (Fig. 6B). HEK-293T cells were transfected with Flag-RNF5 and Myc-STING, then immunoprecipitation (IP) and WB (IB) analysis showed that exogenous expression of RNF5 interacted with exogenous expression of STING (Fig. 6C). Meanwhile, endogenous expression of STING and exogenous expression of RNF5 could also interact with each other in NRCMs (Fig. 6D). This interaction was further confirmed with a GST pull‐down assay (Fig. 6E). Previous studies have shown that STING could regulate pathological cardiac hypertrophy via endoplasmic reticulum stress [17]. We further explored the roles of RNF5 and STING in heart and NRCMs, and the results verified that STING expression was increased in pathological cardiac hypertrophy (Fig. 6F–H). RNF5 deficiency led to up-regulation of STING expression, while RNF5 overexpression had the opposite effect (Fig. 6F–H). Subsequent immunohistochemistry experiment confirmed the results of the RNF5 loss-of-function assay (Fig. 6I). These results suggest that there is a direct interaction between RNF5 and STING, and RNF5 could regulate the expression of STING in pathological cardiac hypertrophy.

### RNF5 promotes K48-linked ubiquitination and proteasomal degradation of STING

**Fig. 7:**
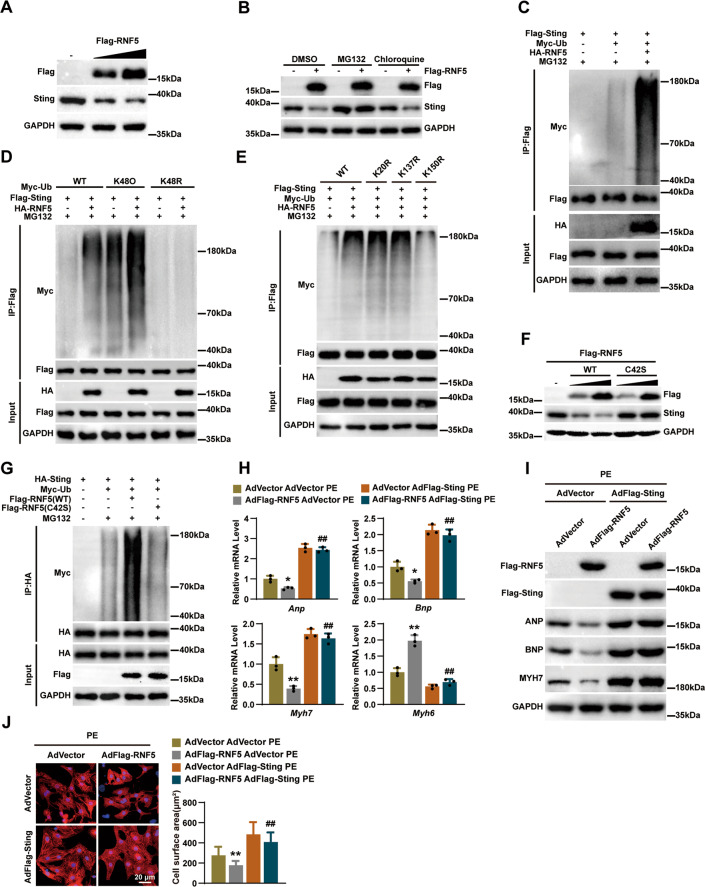
RNF5 promoted STING ubiquitination and degradation. **A** NRCMs were infected with different amounts of AdFlag-RNF5, and the protein expression of STING was detected. **B** NRCMs infected with AdFlag-RNF5 or AdNull were treated with MG132 or chloroquine (CQ) of 25 μM for 6 h before harvest and the protein expression of STING was detected. **C** HEK-293T cells were transfected with Flag-STING, Myc-Ub, and HA-RNF5 plasmids, and the ubiquitination of STING was analyzed by WB. **D** Ubiquitination of STING by RNF5 with the ubiquitin mutants (K48O or K48R). **E** HEK-293T cells were transfected with Flag-STING (WT), Flag-STING (K20R), Flag-STING (K137R), Flag-STING (K150R), Myc-Ub, and HA-RNF5 plasmids, and the ubiquitination of STING was analyzed by WB. **F** After HEK-293T cells were transfected with Flag-RNF5 (WT) and Flag-RNF5 (C42S) plasmids, the protein expression of STING was detected. **G** After HEK-293T cells were transfected with HA-STING, Myc-Ub, Flag-RNF5 (WT), and Flag-RNF5 (C42S) plasmids, MG132 was added 6 h before harvest, and the ubiquitination of STING was analyzed by WB. **H** RT-PCR analysis of the mRNA levels of ANP, BNP, MYH7, and MYH6 in NRCMs infected with AdVector, AdFlag-RNF5, or AdFlag-STING after PE treatment for 24 h. **I** WB analysis of RNF5, ANP, BNP, and MYH7 in NRCMs infected with AdVector, AdFlag-RNF5, or AdFlag-STING after PE treatment for 24 h. **J** NRCMs were infected with AdVector, AdFlag-RNF5, or AdFlag-STING, and then NRCMs were treated with PE for 24 h. Cardiomyocytes were identified by α-actinin staining (red) and nuclei were stained with DAPI (blue). Scale bars: 20 μm. Statistical results for the cell surface area of cardiomyocytes in different groups (*n* = 40 cells per group). For (**H**, **J**) ^*^P < 0.05 or ^**^P < 0.01 versus AdVector AdVector PE, ^##^*P* < 0.01 versus AdFlag-RNF5 AdVector PE. Data were presented as the mean ± SD. Statistical analysis was carried out by one-way ANOVA.

E3 ubiquitin ligase can transfer ubiquitin from E2 ubiquitin-conjugating to host protein, thereby labeling substrates for proteasome digestion [18]. RNF5 is one of the E3 ubiquitin ligases, and we, therefore, postulated that RNF5 regulated the stabilization of STING. After we infected NRCMs with AdFlag-RNF5, as shown in Fig. 7A, the expression of STING protein was significantly downregulated with the increasing amount of AdFlag-RNF5 infection. Then, we found that the addition of a 26 S proteasome inhibitor (MG132), but not a lysosome inhibitor (chloroquine [CQ]), could abolish RNF5-induced degradation of STING (Fig. 7B), which indicated that RNF5 facilitated the proteasomal degradation of STING. Further experiments showed that RNF5 regulated the stabilization of STING by ubiquitination of STING (Fig. 7C). K48-linked polyubiquitin chains are sufficient to target the degradation of a substrate protein in ubiquitin-mediated proteolysis [19]. The ubiquitination assay results revealed that RNF5 promoted the addition of K48-linked polyubiquitin chains to degrade STING, and when K48 ubiquitination sites were mutated, RNF5-mediated ubiquitination of STING was abolished (Fig. 7D). Previous studies indicated that the ubiquitin sites of STING might be located at 1-160 [ref. 16]. By mutating lysine residues of K20, K137, and K150 in this region of STING to arginine, we found that RNF5-mediated STING ubiquitination was canceled only when K150 was mutated to arginine (Fig. 7E). RNF5C42S, in which the Cys42 in the ring-finger domain is mutated to serine, is well accepted as an inactive catalytic mutant of RNF5 [ref. 9], which loses the ability to ubiquitinate and degrade STING (Fig. 7F, G). To explore whether the function of RNF5 regulated cardiac hypertrophy depending on STING, we overexpressed RNF5 and STING, respectively, or simultaneously in NRCMs. RT-PCR, WB, and immunofluorescence confirmed that overexpression of STING abolished the regulation of RNF5 in cardiac hypertrophy (Fig. 7H–J). Collectively, these data suggest that RNF5 alleviates pathological cardiac hypertrophy through K48-linked ubiquitination and proteasomal degradation of STING.

## Discussion

In this study, we revealed the function of RNF5 in pathological cardiac hypertrophy. We constructed cellular and animal models of cardiac hypertrophy and found that the expression of RNF5 was increased in these models. The loss-of-function research showed that RNF5 deficiency exacerbated cardiac remodeling and dysfunction, reactivation of fetal gene expression, fibrosis, and significant activation of the NF-κB signaling. Whereas the gain-of-function studies demonstrated that overexpression of RNF5 attenuated cardiomyocyte hypertrophy. We further investigated the molecular mechanism of RNF5 in the development of pathological cardiac hypertrophy. The protein–protein interaction experiment confirmed the interaction between STING and RNF5. Rescue experiments showed that overexpression of STING could attenuate the effect of RNF5 on alleviating cardiac hypertrophy. Therefore, our study suggests that targeting RNF5 may be a promising strategy for the treatment of pathological cardiac hypertrophy.

The essence of cardiac hypertrophy is the increase of protein content in cardiomyocytes, and the ubiquitin-proteasome system (UPS) plays an important role in regulating the dynamic balance of protein metabolism in cardiomyocytes [20]. RNF5 locates in the proximal region of the major histocompatibility complex (MHC) on chromosome 6 [ref. 21]. The ring-finger domain of RNF5 shows its activity of E3 ubiquitin ligase. Retention of misfolded Pendrin mutants in the endoplasmic reticulum is considered the main pathological mechanism of Pendred syndrome, and RNF5 has significant effects on Pendrin protein degradation [22]. Young et al. found that in the endoplasmic reticulum stress (ER stress) response of breast cancer cells induced by paclitaxel, RNF5 inhibited the mTOR signaling pathway through binding and ubiquitination of SLC1A5/38A2, thus regulating the response of breast cancer to ER stress-induced chemotherapy [23]. Kuang et al. found that under normal physiological conditions, RNF5 could combine AGT4B to promote the degradation of AGT4B and thus regulate autophagy [24]. However, the potential association between RNF5 and pathological cardiac hypertrophy has not been explored.

Proinflammatory cytokines such as tumor necrosis factor (TNF-α), interleukin 1β (IL-1β), and interleukin 6 (IL-6) can inhibit myocardial contraction, promote myocardial fibrosis, and increase collagen synthesis in pathological cardiac hypertrophy [14, 25, 26]. Previous studies have demonstrated that RNF5 inhibits the activation of IRF3 and NF-κB signaling pathways by mediating the ubiquitination and degradation of STING triggered by viral infection to avoid excessive antiviral responses [16]. By infecting AdFlag-RNF5 in NRCMs and detecting the protein expression level of STING, we found that RNF5 was associated with regulating STING in cardiac myocytes. The correlation between RNF5 and STING was further verified by protein–protein interaction experiments. There are two main protein degradation systems in eukaryotic cells, the autophagy-lysosome system and the ubiquitin-proteasome system [27]. The application of a 26 S proteasome inhibitor (MG132) in NRCMs infected with AdFlag-RNF5 prevented RNF5 from degrading STING. The main ubiquitin chain type for proteasome pathway degradation of proteins is the Lys48 linkage [28]. RNF5-mediated STING ubiquitination was eliminated when the K48 ubiquitin site was mutated. By mutating the ubiquitination site of STING, we found that RNF5 targeted STING at K150 for ubiquitination. The C42 residue in the RING domain is required for RNF5 to exert ubiquitin ligase activity [9]. When the Cys42 was mutated to serine, RNF5 lost the ability to ubiquitinate and degrade STING. Based on these observations, we speculate that RNF5 is a negative regulator of STING-mediated signaling.

There are some limitations to the study. Our results confirmed the role of RNF5 in regulating cardiac hypertrophy by using RNF5 knockout mice, but we did not perform RNF5 overexpression in vivo, which would better verify our results. In our study, we focused on the regulation of RNF5 on cardiomyocytes. We found that overexpression and inhibition of RNF5 could significantly inhibit and promote myocardial hypertrophy induced by PE stimulation, respectively. We also observed that RNF5 is involved in fibrosis and inflammation in cardiac hypertrophy, but whether RNF5 is involved by directly modulating the function of other cell types in the heart or by regulating cardiomyocyte paracrine function requires further study.

In conclusion, our study suggests that RNF5 is one of the negative regulators of pathological cardiac hypertrophy. RNF5 inhibits the development of cardiac hypertrophy by targeting STING and promoting its K48- linked ubiquitination-mediated degradation. These results provide a new perspective for studying the role of ER-associated protein in the pathogenesis of pathological cardiac hypertrophy.

## Supplementary information

Reproducibility Checklist

Supplementary tables

Original western blots

## Data Availability

The datasets generated and/or analyzed during the current study are available from the corresponding author on reasonable request.
