# Supplementary material for: E3 ubiquitin ligase RNF5 attenuates pathological cardiac hypertrophy through STING
Source: Cell Death Dis. 2022 Oct 21;13(10):889. doi: 10.1038/s41419-022-05231-8 (PMC9587004; doi:10.1038/s41419-022-05231-8)
Supplement: Supplementary file 2 — Supplementary tables [file 41419_2022_5231_MOESM2_ESM.docx]

Supplementary table 1

| Anbibody | Catalogue number | Manufacturer | Source of species | Dilution |
| --- | --- | --- | --- | --- |
| RNF5 | A8351 | ABclonal | rabbit | 1:1000 |
| ANP | 27426-1-AP | Proteintech | rabbit | 1:1000 |
| BNP | A2179 | ABclonal | rabbit | 1:1000 |
| MYH7 | 22280-1-AP | Proteintech | rabbit | 1:1000 |
| CollagenIα1 | A16891 | ABclonal | rabbit | 1:1000 |
| CollagenⅢα1 | A0817 | ABclonal | rabbit | 1:1000 |
| CTGF | sc-14939 | santa cruz | rabbit | 1:1000 |
| p-IKKβ | 2697 | CST | rabbit | 1:1000 |
| IKKβ | A0714 | ABclonal | rabbit | 1:1000 |
| p-IκBα | 2859 | CST | rabbit | 1:1000 |
| IκBα | 4814 | CST | rabbit | 1:1000 |
| p-p65 | 3033 | CST | rabbit | 1:1000 |
| p65 | 8242 | CST | rabbit | 1:1000 |
| Flag | M185-3LL | MBL | rabbit | 1:1000 |
| HA | M180-3 | MBL | rabbit | 1:1000 |
| Myc | M047-3 | MBL | rabbit | 1:1000 |
| Sting | 19851-1-ap | Proteintech | rabbit | 1:1000 |
| GAPDH | 60004-1-Ig | Proteintech | rabbit | 1:5000 |

Supplementary table 2

| Gene name | Forward primer (mouse) | Reverse primer (mouse) |
| --- | --- | --- |
| *RNF5* | GAATGCCCGGTGTGTAAAGC | GGGGTGGAGTTTTCAATCTGG |
| *Anp* | TCGGAGCCTACGAAGATCCA | TTCGGTACCGGAAGCTGTTG |
| *Bnp* | GAAGGACCAAGGCCTCACAA | TTCAGTGCGTTACAGCCCAA |
| *Myh7* | CAACCTGTCCAAGTTCCGCA | TACTCCTCATTCAGGCCCTTG |
| *Myh6* | TCTGCCTACCTTATGGGGCT | CGTACACTGACTTGGCCAGT |
| *Collagen Iα1* | TGCTAACGTGGTTCGTGACCGT | ACATCTTGAGGTCGCGGCATGT |
| *Collagen IIIα1* | ACGTAAGCACTGGTGGACAG | CCGGCTGGAAAGAAGTCTGA |
| *CollagenⅧα1* | GCAACCAGGAGCAAAAGGTG | ATGCATACCTGGAGGACCCT |
| *Ctgf* | TGACCCCTGCGACCCACA | TACACCGACCCACCGAAGACACAG |
| *IL6* | TAGTCCTTCCTACCCCAATTTCC | TTGGTCCTTAGCCACTCCTTC |
| *IL1β* | CCGTGGACCTTCCAGGATGA | GGGAACGTCACACACCAGCA |
| *Tnfα* | CATCTTCTCAAAATTCGAGTGACAA | TGGGAGTAGACAAGGTACAACCC |
| *Gapdh* | ACTCCACTCACGGCAAATTC | TCTCCATGGTGGTGAAGACA |
|  |  |  |
| Gene name | Forward primer (rat) | Reverse primer (rat) |
| *RNF5* | TATGGTCGAGGGAGCCAGAA | TGAAATCCCCCTGCATCACC |
| *Anp* | AAAGCAAACTGAGGGCTCTGCTCG | TTCGGTACCGGAAGCTGTTGCA |
| *Bnp* | TGCCCCAGATGATTCTGCTC | TGTAGGGCCTTGGTCCTTTG |
| *Myh6* | CTCCAGGGGTGATGGACAAC | CGATACCTCTGCCGGAAGTC |
| *Myh7* | AGTTCGGGCGAGTCAAAGATG | CAGGTTGTCTTGTTCCGCCT |
| *Gapdh* | CAGTGCCAGCCTCGTCTCAT | AGGGGCATCCACAGTCTTC |

Supplementary table 3

| Gene | Primer sequence(5’-3’) | |
| --- | --- | --- |
| AdshRNF5 | F | CCGGGCGACCTTCGAATGTAATATACTCGAGTATATTACATTCGAAGGTCGCTTTTTG |
|  | R | AATTCAAAAAGCGACCTTCGAATGTAATATACTCGAGTATATTACATTCGAAGGTCGC |
| AdSting | F | GGCTAGCGATATCGGATCCGCCACCATGCCATACTCCAACCTG CA |
|  | R | CGTCCTTGTAATCACTAGTGATGAGGTCCGTGCGGAG |

Supplementary table 4

| Gene | Primer sequence(5’-3’) | |
| --- | --- | --- |
| Flag-RNF5 | F | TCGGGTTTAAACGGATCCATGGCAGCAGCGGAGGAGGA |
|  | R | GGGCCCTCTAGACTCGAGTCAAATACTGAGCAGCCAAA |
| Myc-Sting | F | TCGGGTTTAAACGGATCCATGCCCCACTCCAGCCTG |
|  | R | GGGCCCTCTAGACTCGAGTCAAGAGAAATCCGTGCGGAG |
| Flag-Sting | F | TCGGGTTTAAACGGATCCATGCCCCACTCCAGCCTG |
|  | R | GGGCCCTCTAGACTCGAGTCAAGAGAAATCCGTGCGGAG |
| HA-RNF5 | F | TCGGGTTTAAACGGATCCATGGCAGCAGCGGAGGAGGA |
|  | R | GGGCCCTCTAGACTCGAGTCAAATACTGAGCAGCCAAA |
| HA-Sting | F | TCGGGTTTAAACGGATCCATGCCCCACTCCAGCCTG |
|  | R | GGGCCCTCTAGACTCGAGTCAAGAGAAATCCGTGCGGAG |
| Flag-RNF5 (C42S) | F | CTGTGGTCAGTGTGAGTGGCCACCTGTAC |
|  | R | GTACAGGTGGCCACTCACACTGACCACAG |
| Flag-Sting(K20R) | F | CGGGGCCCAGAGGGCAGCCTTGG |
|  | R | CCAAGGCTGCCCTCTGGGCCCCG |
| Flag-Sting(K137R) | F | CCTGGGCCTCAGGGGCCTGGCCC |
|  | R | GGGCCAGGCCCCTGAGGCCCAGG |
| Flag-Sting(K150R) | F | GCAGTGTGTGAAAGAGGGAATTTCAAC |
|  | R | GTTGAAATTCCCTCTTTCACACACTGC |
| GST-HA-STING | F | TCGGGTTTAAACGGATCCATGTCCGCCGCAGACGAGGT |
| GST-HA-STING | R | GGGCCCTCTAGACTCGAGTTACTTCCCGTAGAACTTTTTGTTGAG |
| GST-HA-RNF5 | F | TCGGGTTTAAACGGATCCATGGCAGCAGCGGAGGAGGA |
| GST-HA-RNF5 | R | GGGCCCTCTAGACTCGAGTCAAATACTGAGCAGCCAAA |
