# Supplementary material for: E3 ubiquitin ligase RNF5 attenuates pathological cardiac hypertrophy through STING
Source: Cell Death Dis. 2022 Oct 21;13(10):889. doi: 10.1038/s41419-022-05231-8 (PMC9587004; doi:10.1038/s41419-022-05231-8)
Supplement: Supplementary file 3 — Original western blots [file 41419_2022_5231_MOESM3_ESM.pdf]

Figure1-C

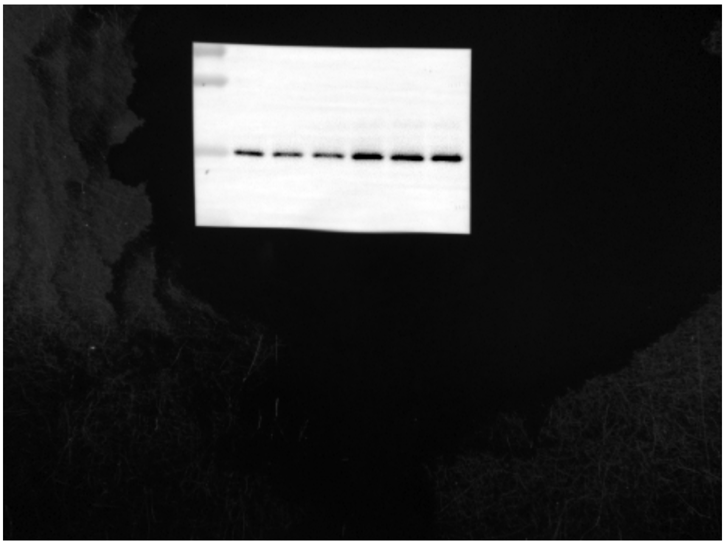

ANP

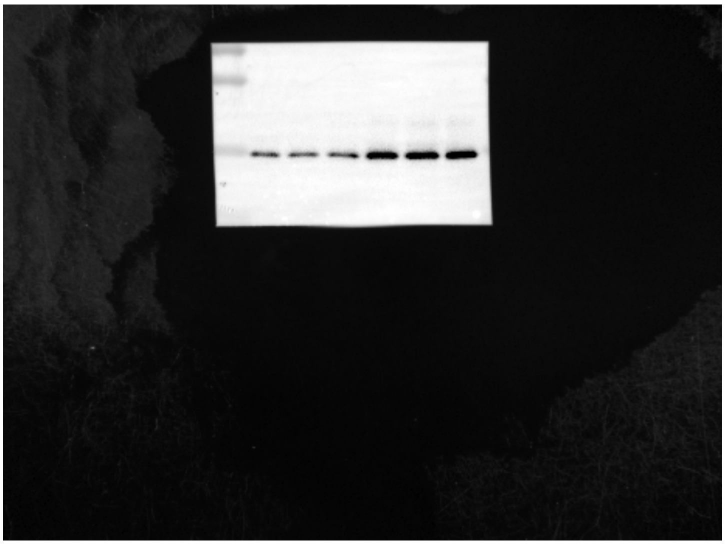

BNP

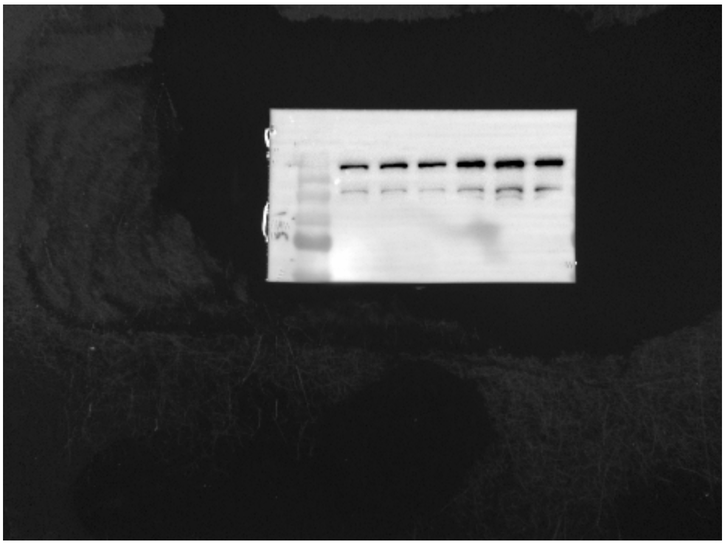

MYH7

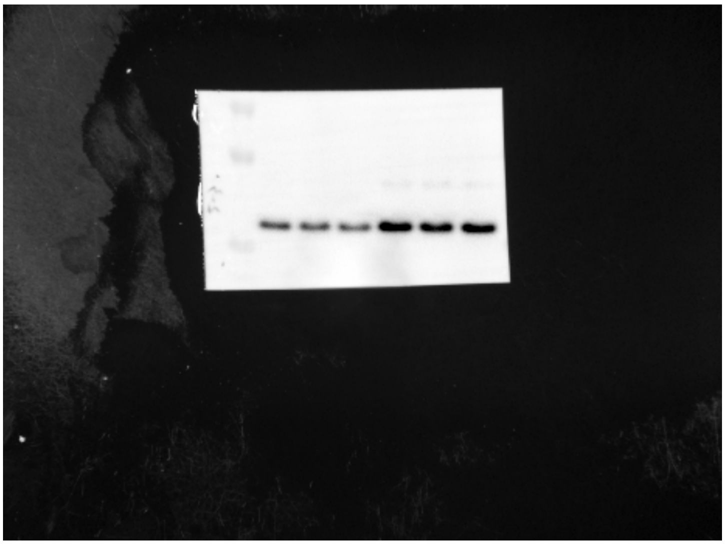

RNF5

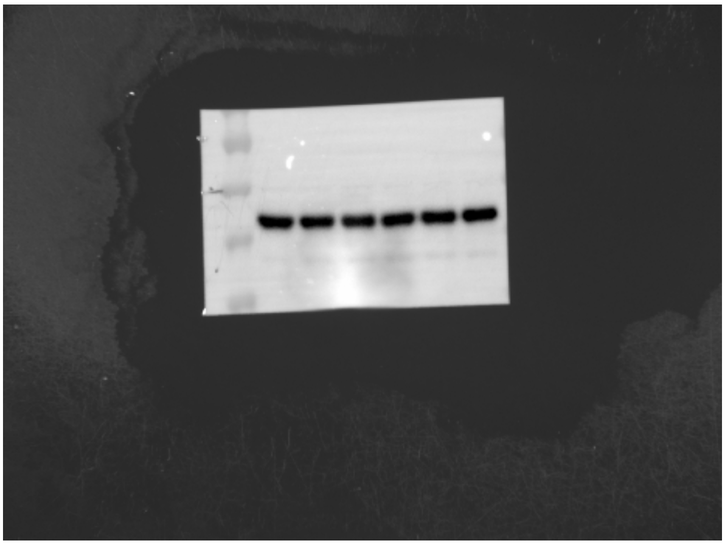

GAPDH

Figure1-E

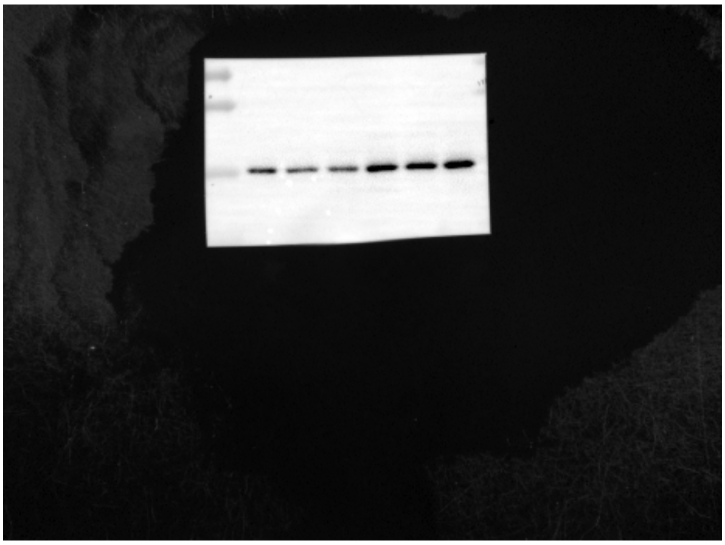

ANP

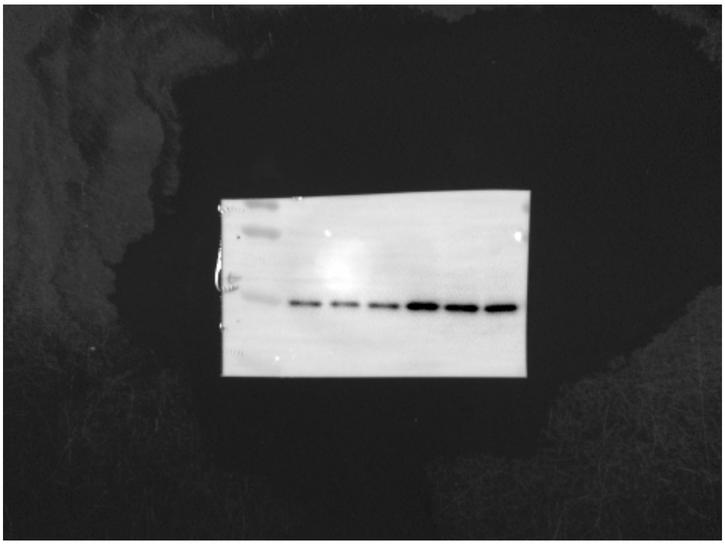

BNP

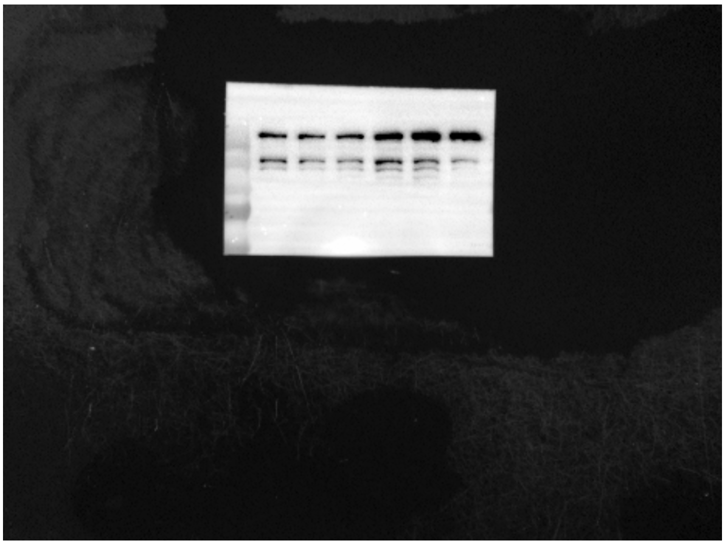

MYH7

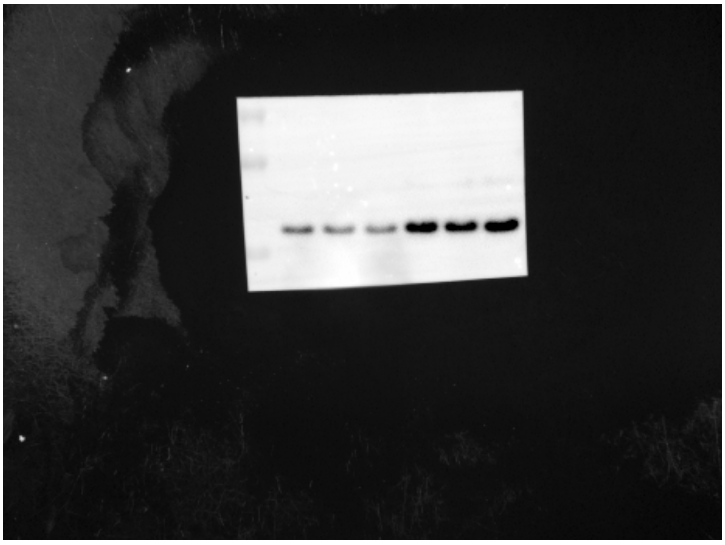

RNF5

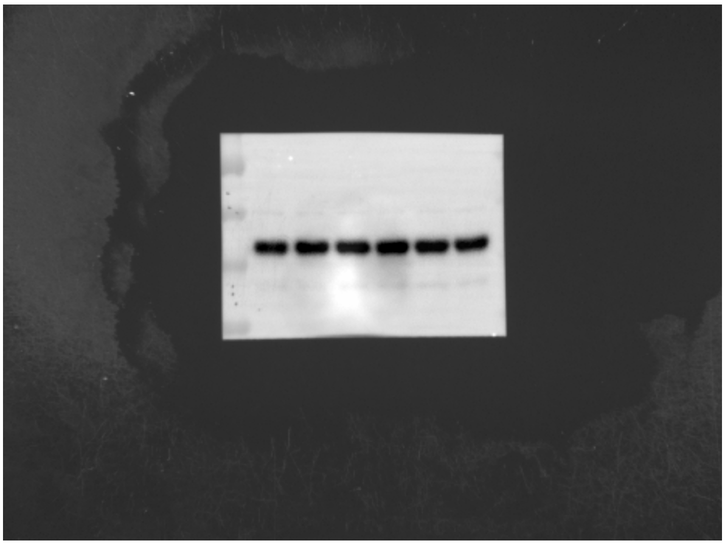

GAPDH

Figure2-A

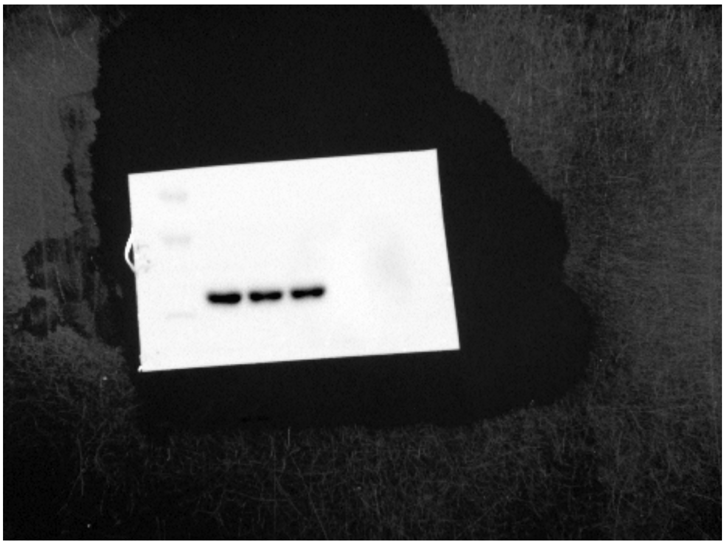

RNF5

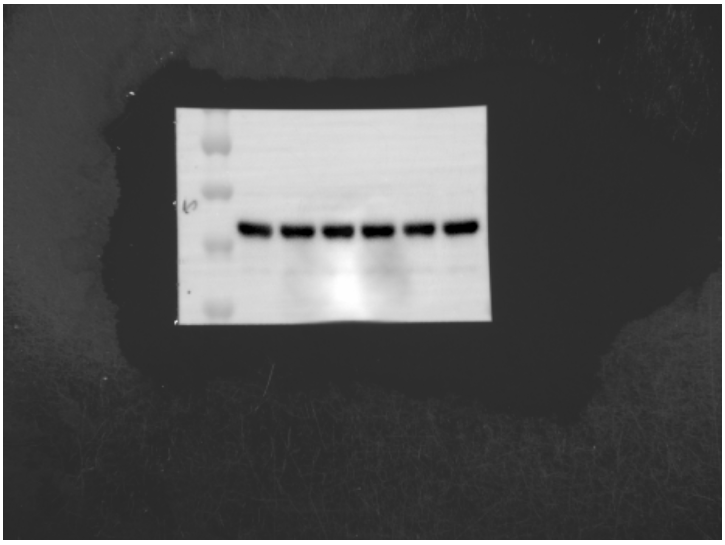

GAPDH

Figure2-G

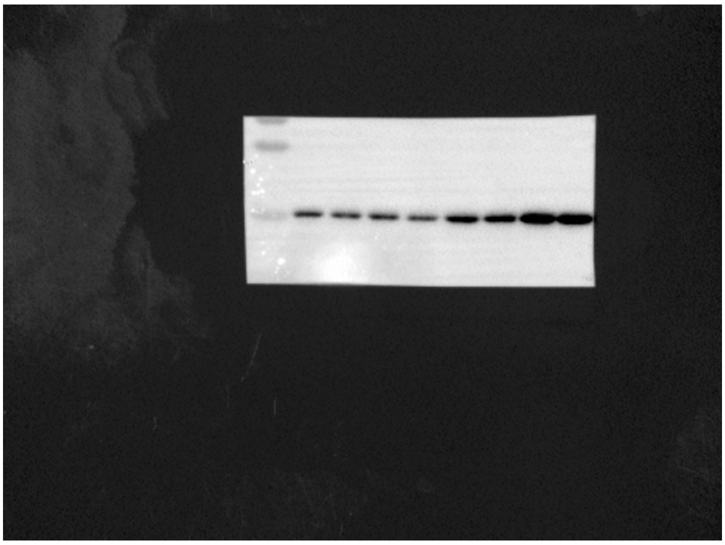

ANP

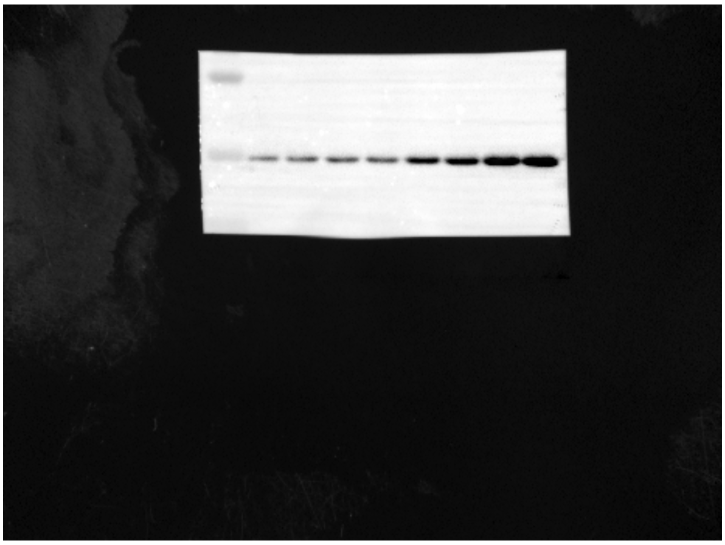

BNP

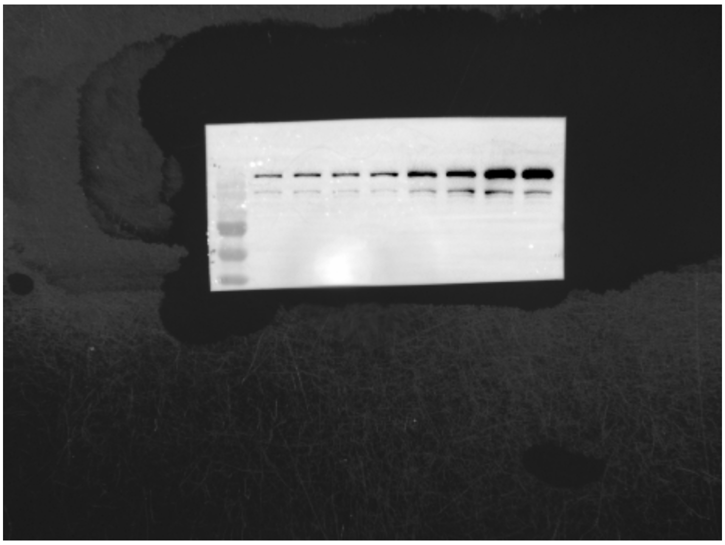

MYH7

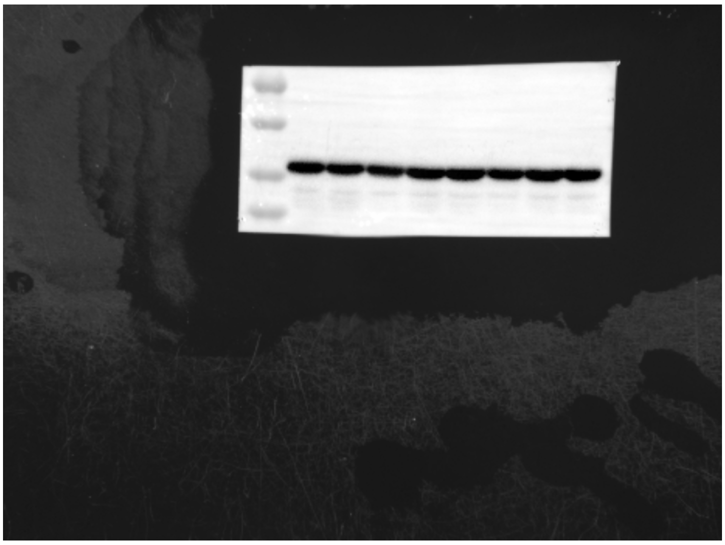

GAPDH

Figure3-C

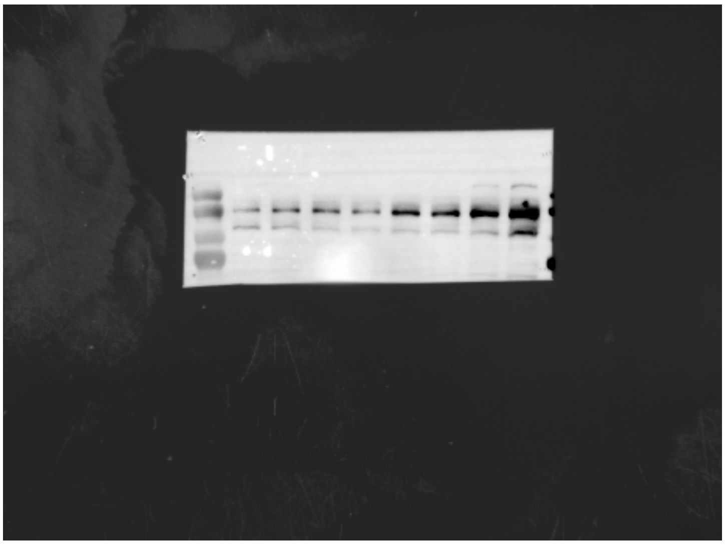

COL1A1

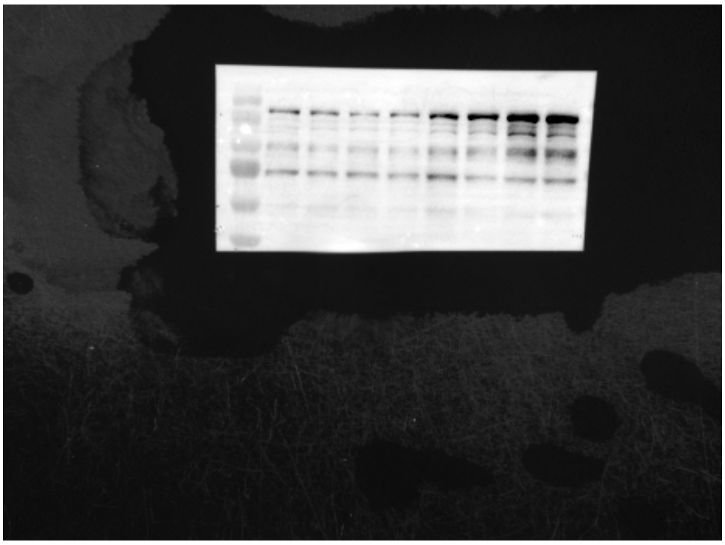

COL3A1

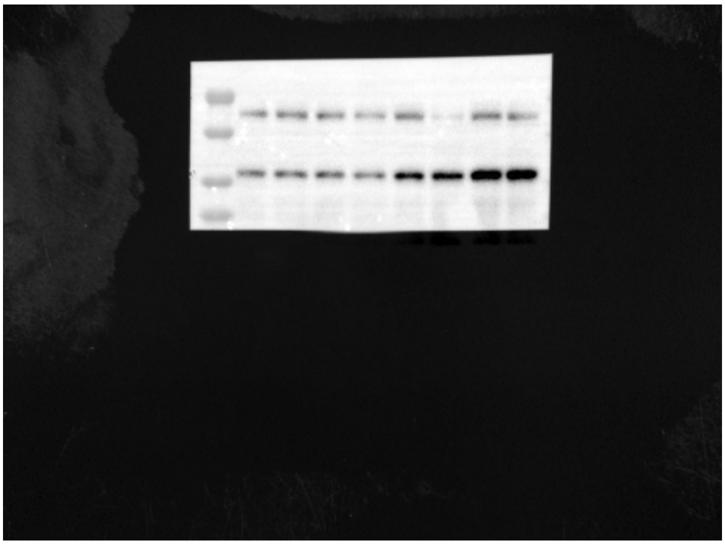

CTGF

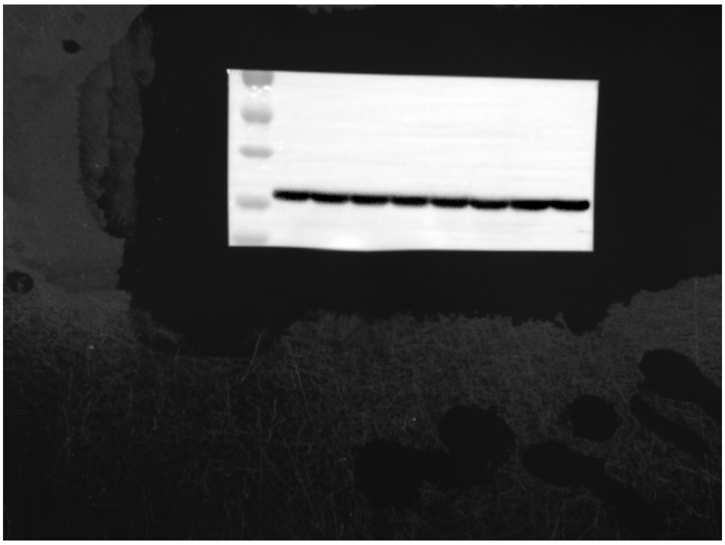

GAPDH

Figure3-E

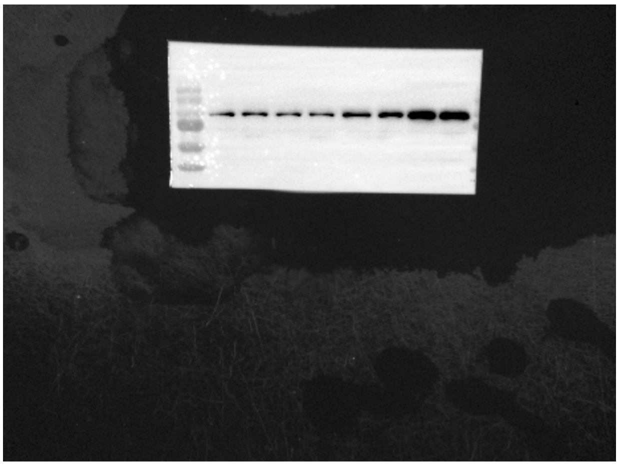

P-IKKβ

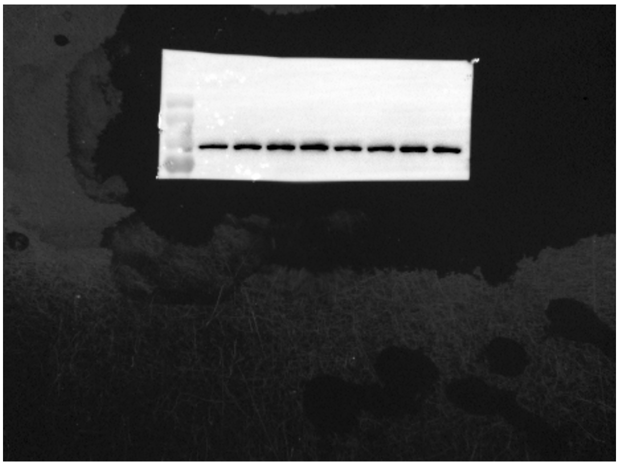

IKKβ

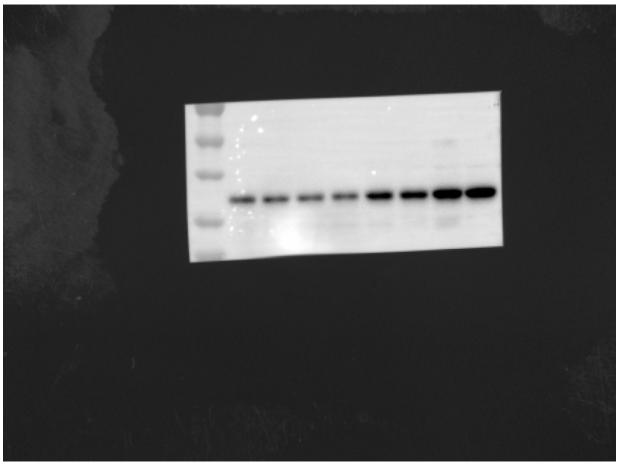

P-IKBα

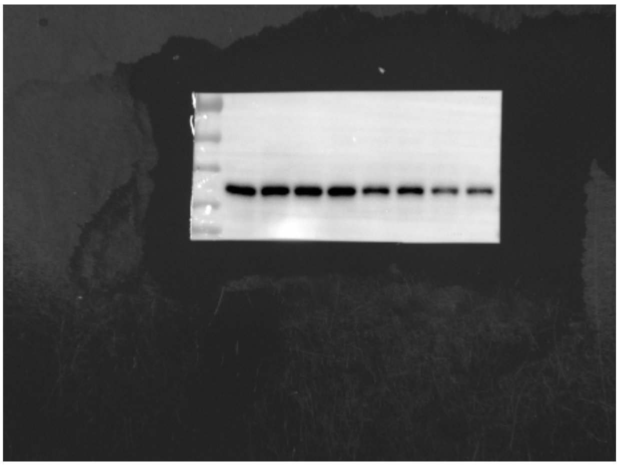

IKBα

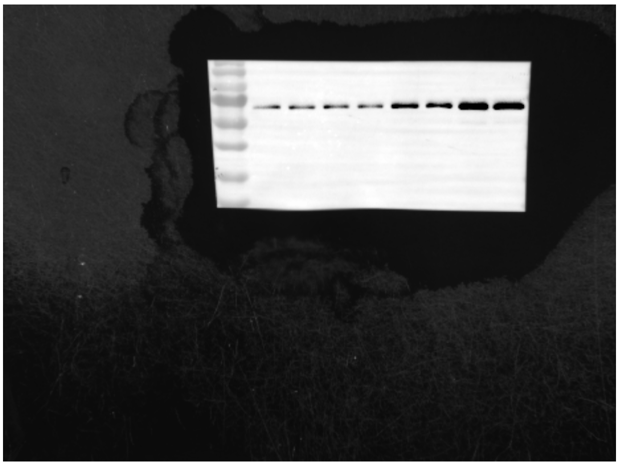

P-P65

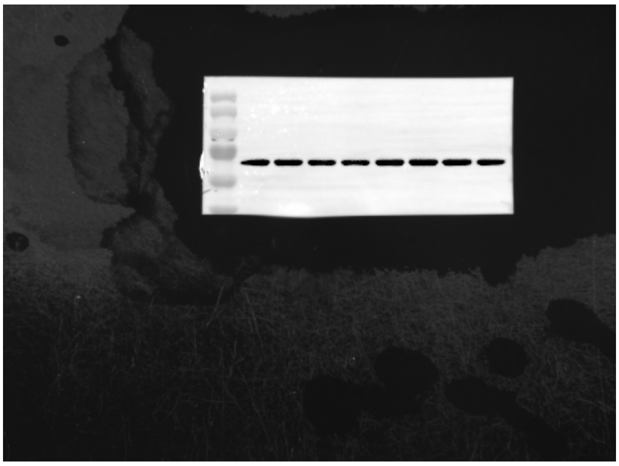

P65

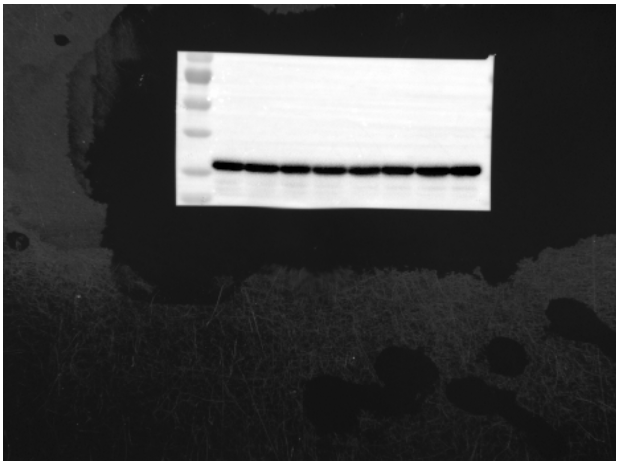

GAPDH

Figure4-A

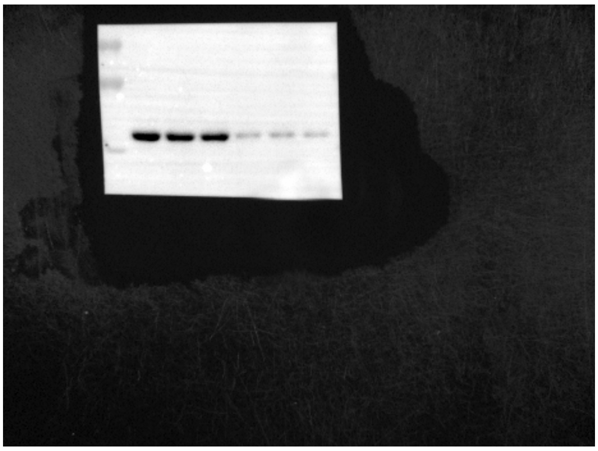

RNF5

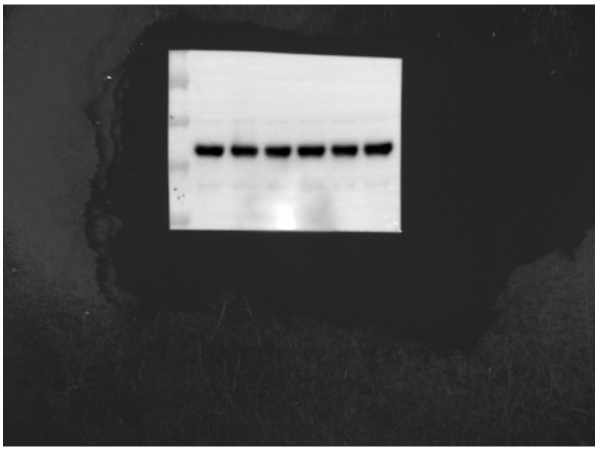

GAPDH

Figure4-D

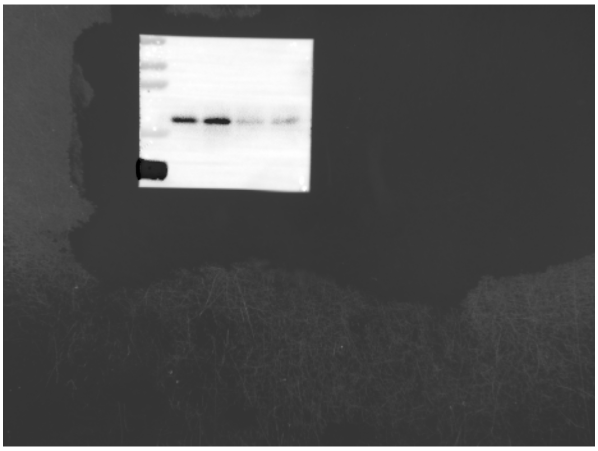

RNF5

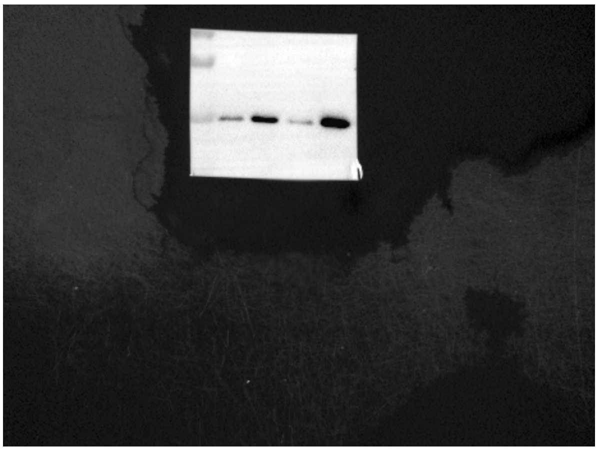

ANP

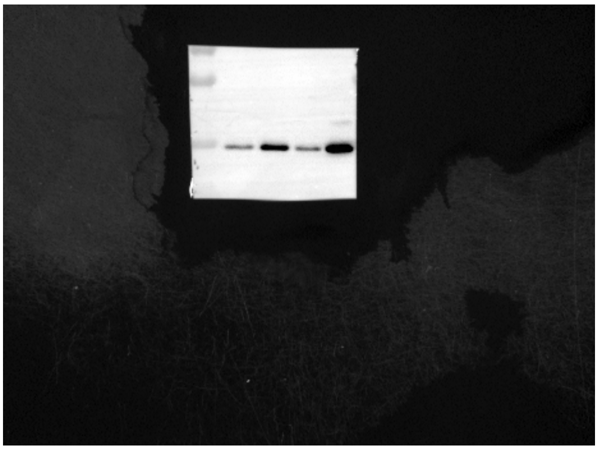

BNP

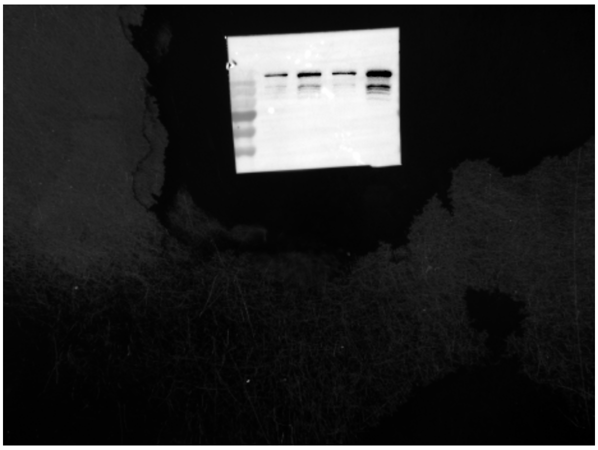

MYH7

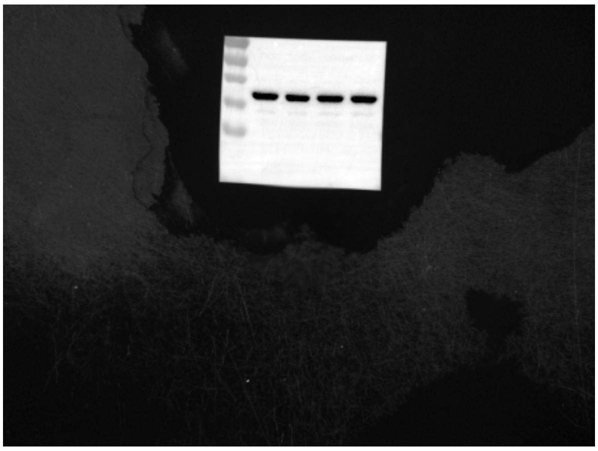

GAPDH

Figure4-E

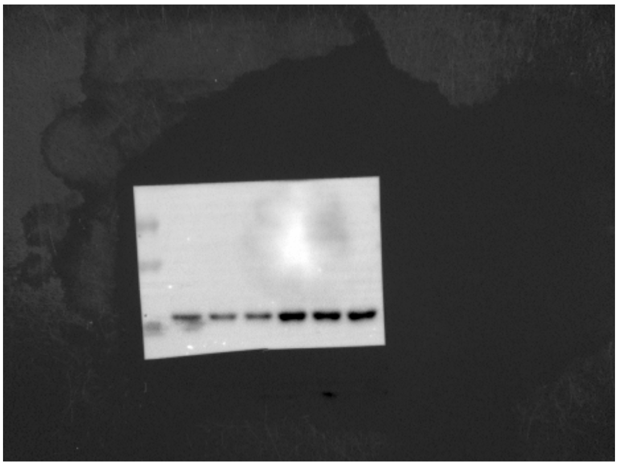

FLAG

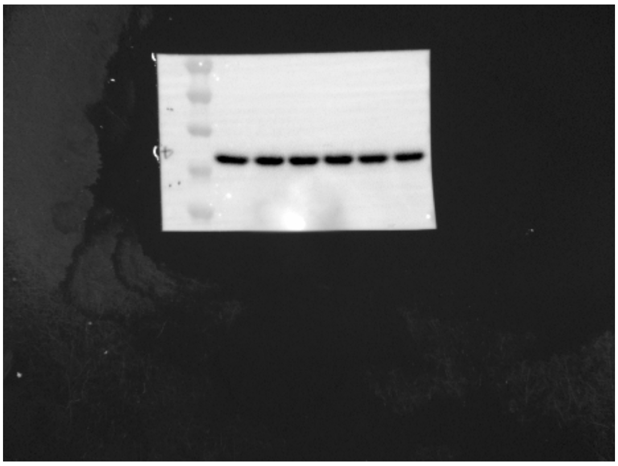

GAPDH

Figure4-H

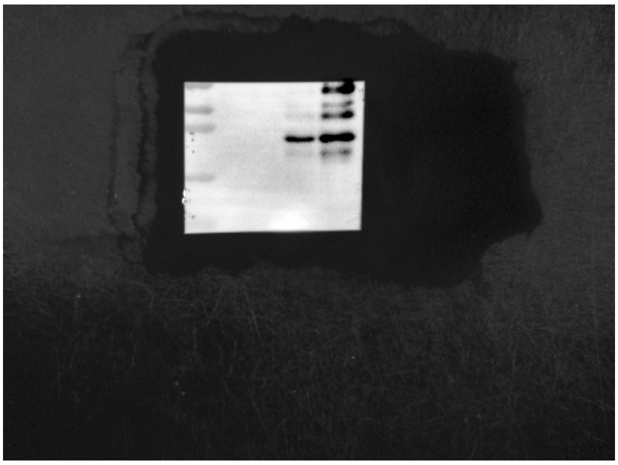

FLAG

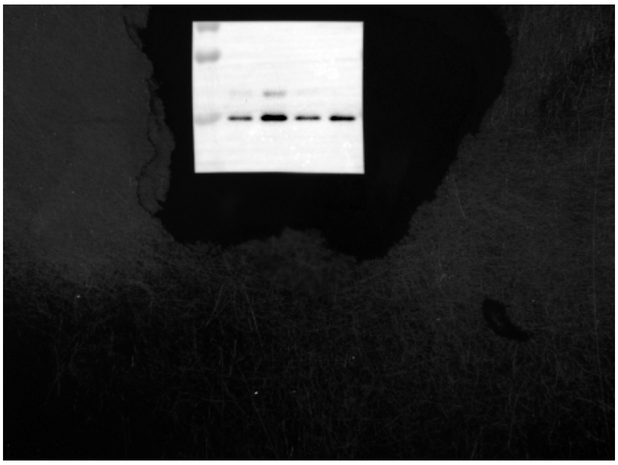

ANP

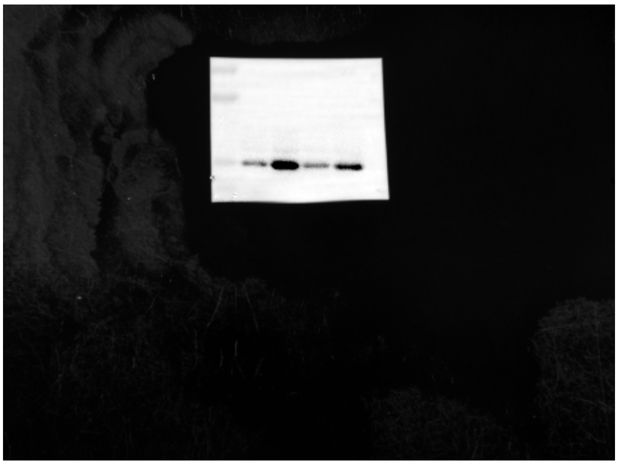

BNP

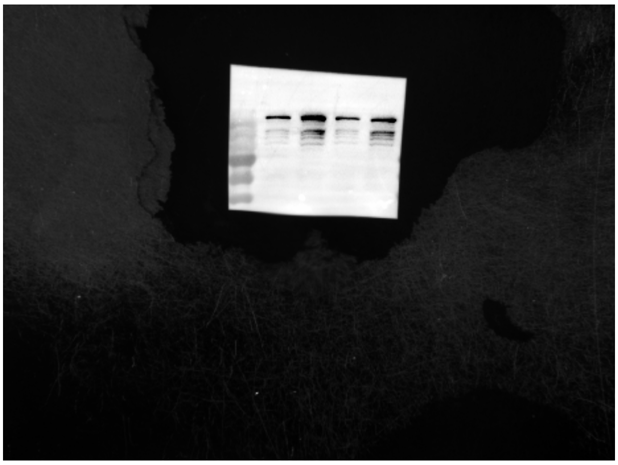

MYH7

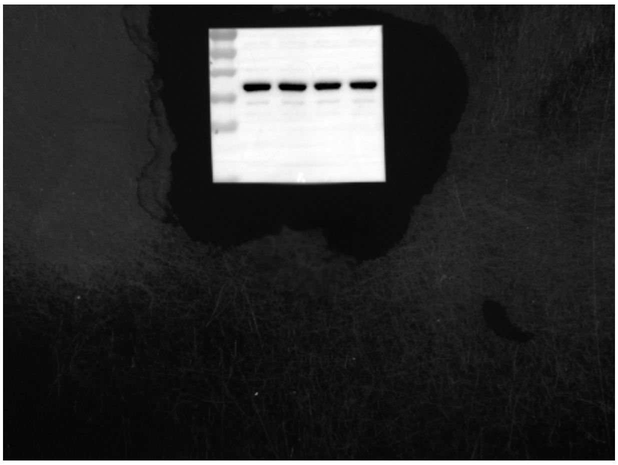

GAPDH

Figure6-C-left

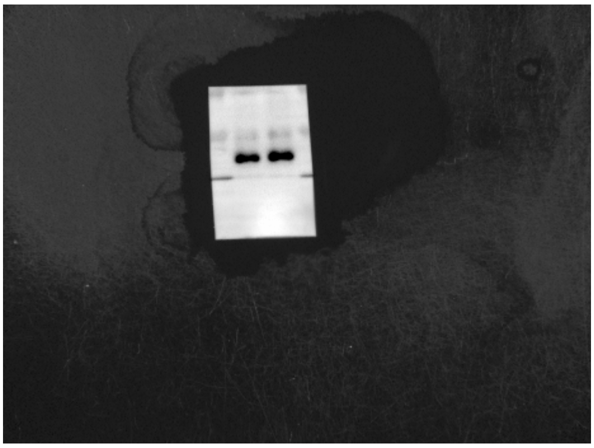

IN FLAG

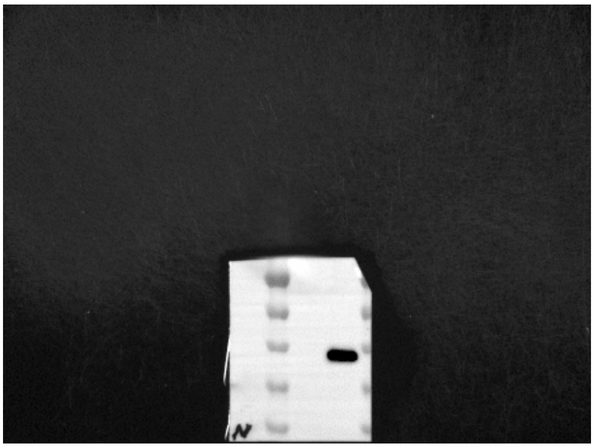

IN MYC

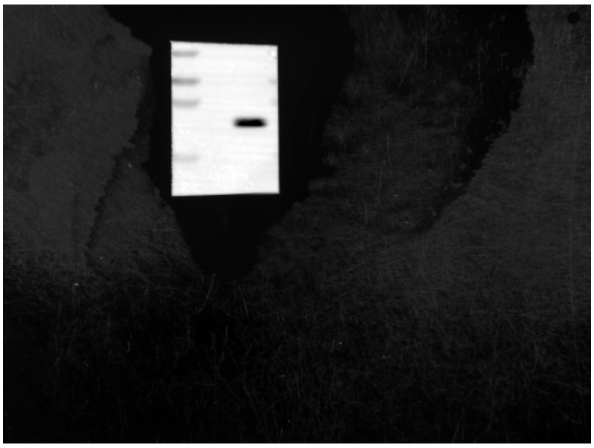

IP FLAG

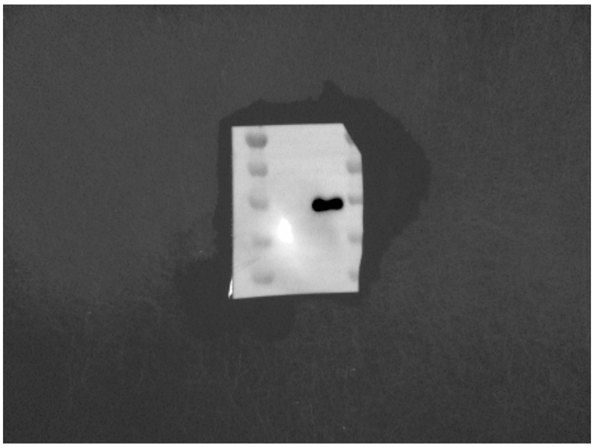

IP MYC

Figure6-C-right

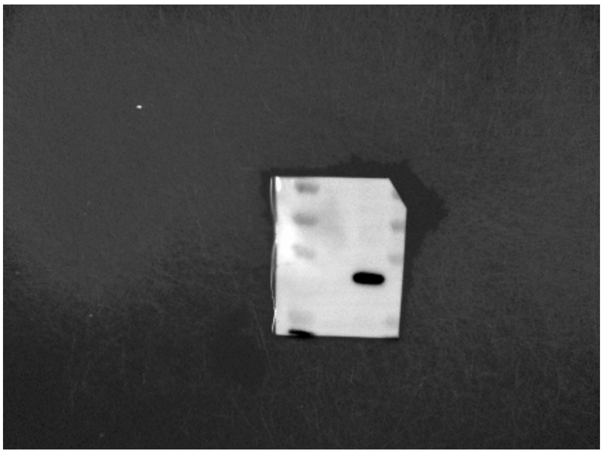

IN FLAG

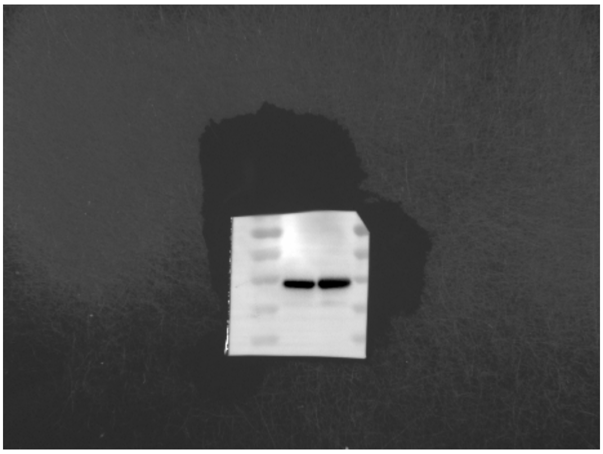

IN MYC

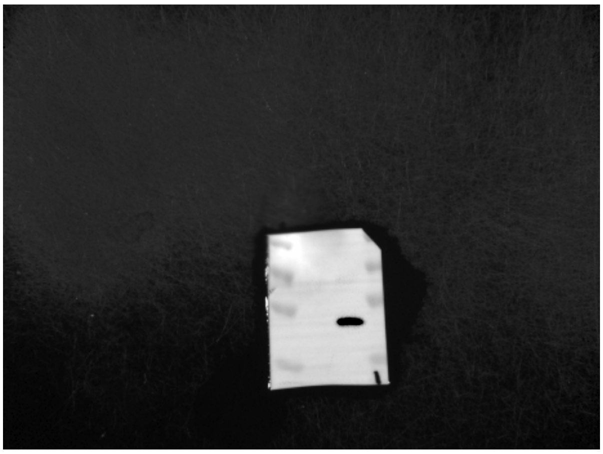

IP FLAG

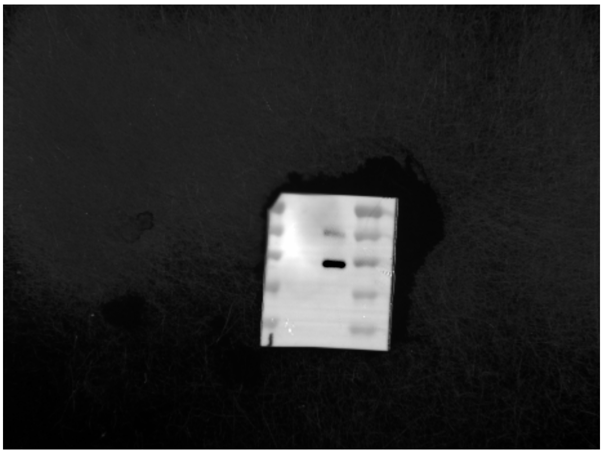

IP MYC

Figure6-D

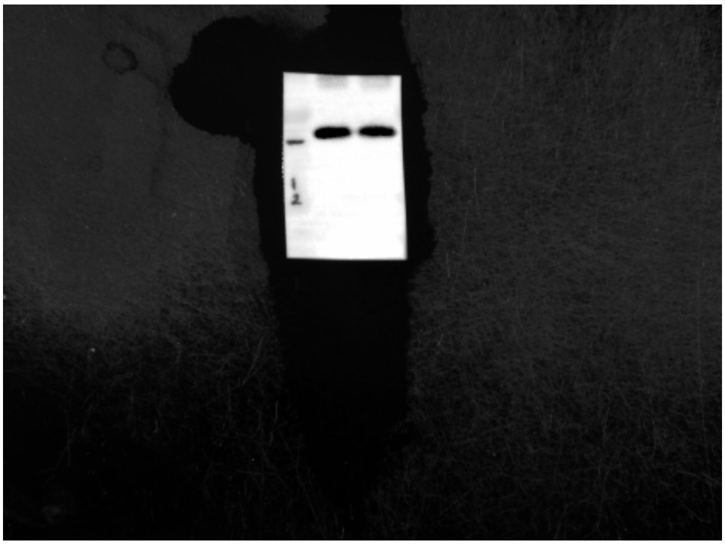

IN FLAG

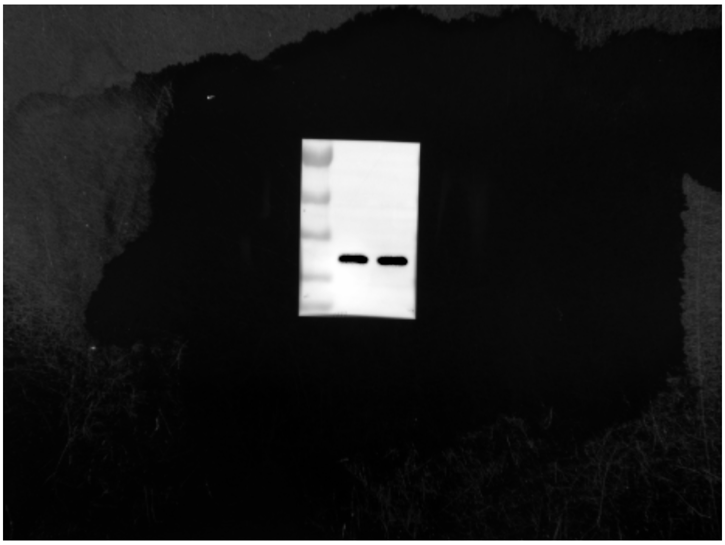

IN STING

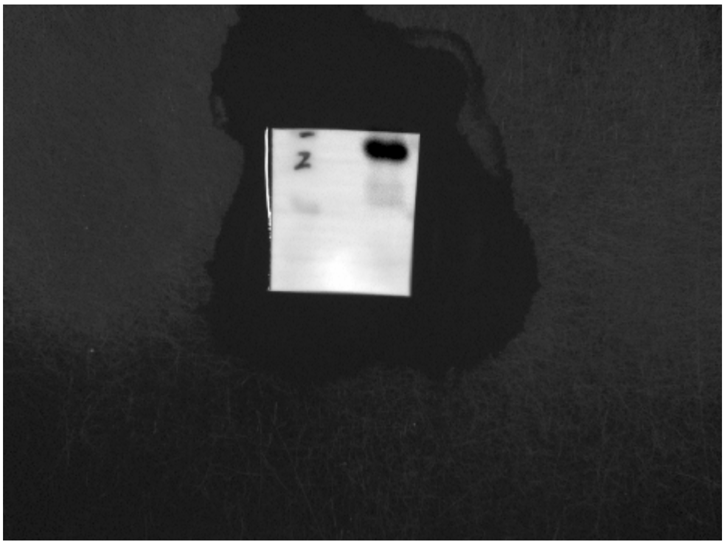

IP FLAG

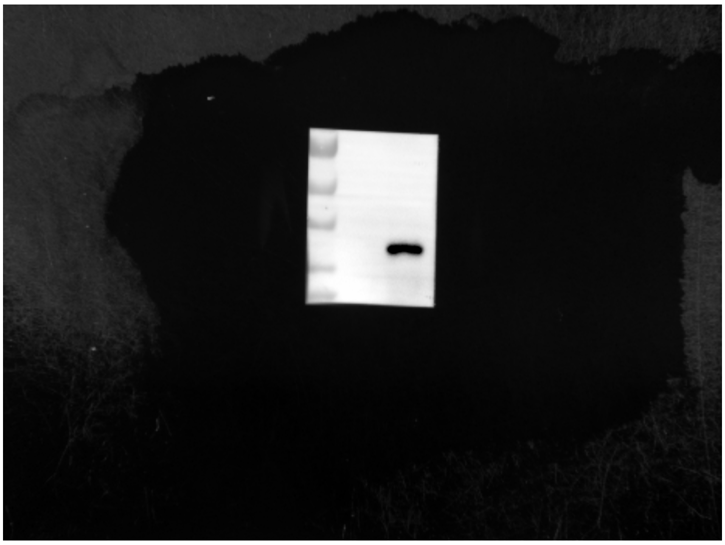

IP STING

Figure6-E-left

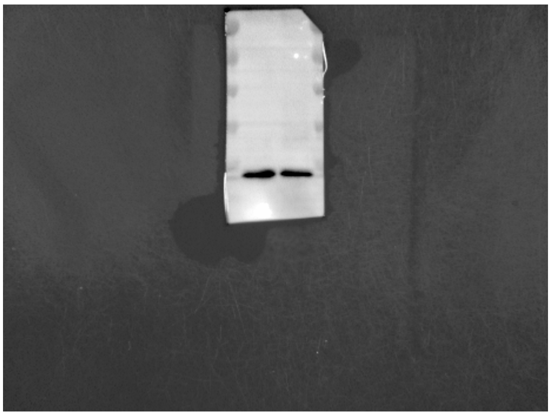

IN FLAG

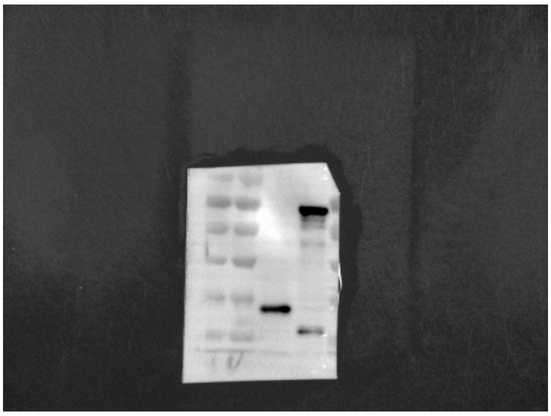

IN HA

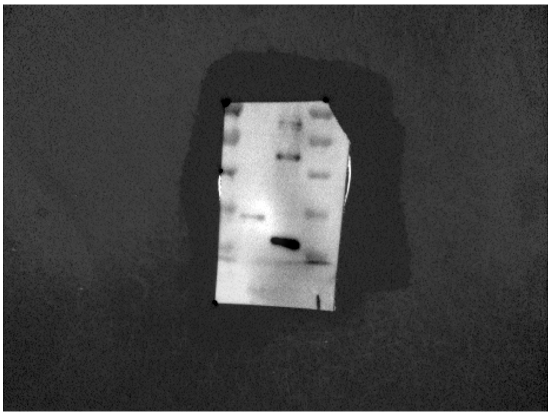

IP FLAG

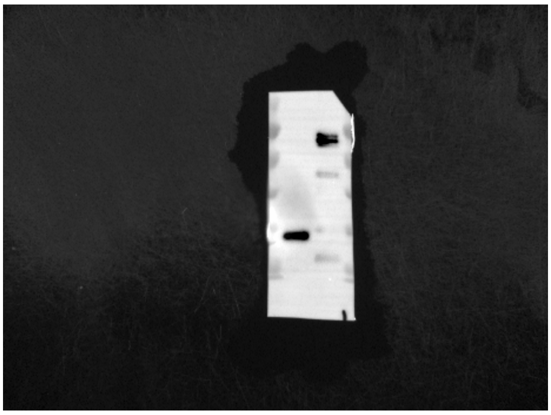

IP HA

Figure6-E-right

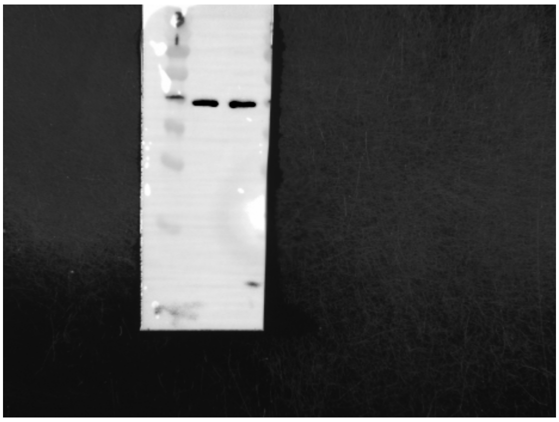

IN FLAG

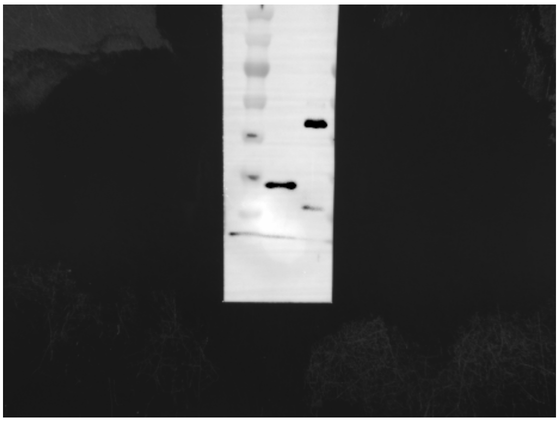

IN HA

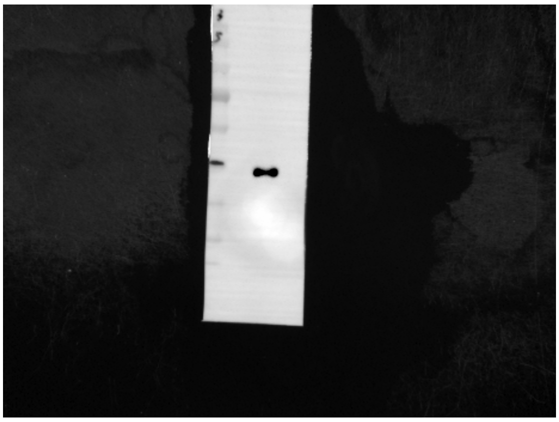

IP FLAG

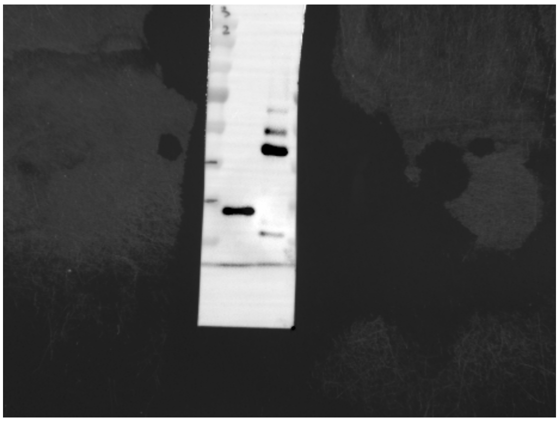

IP HA

Figure6-F

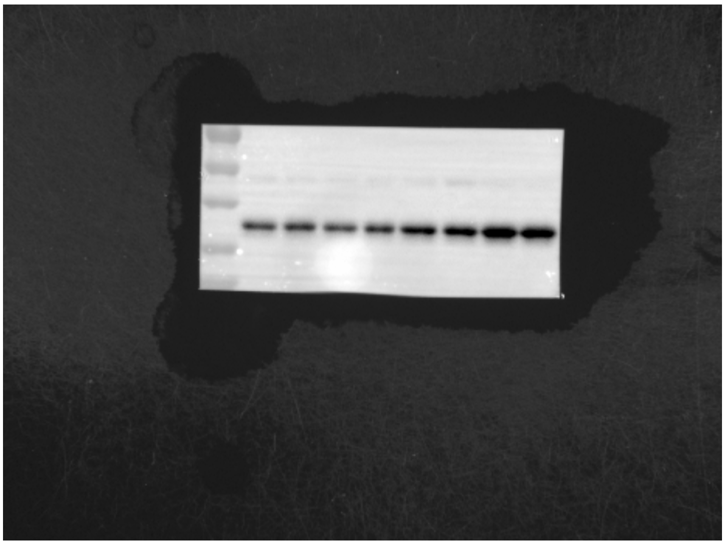

STING

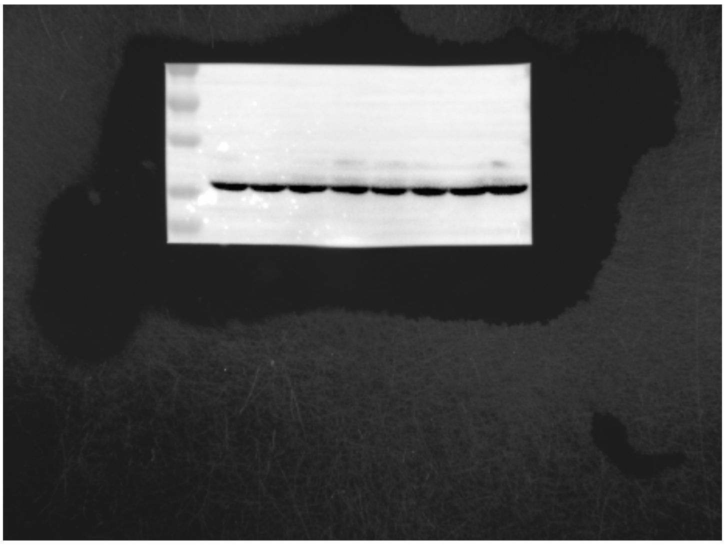

GAPDH

Figure6-G

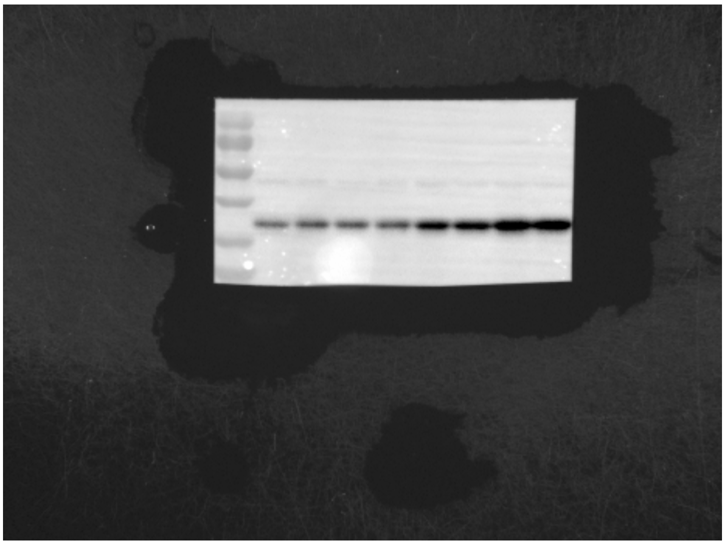

STING

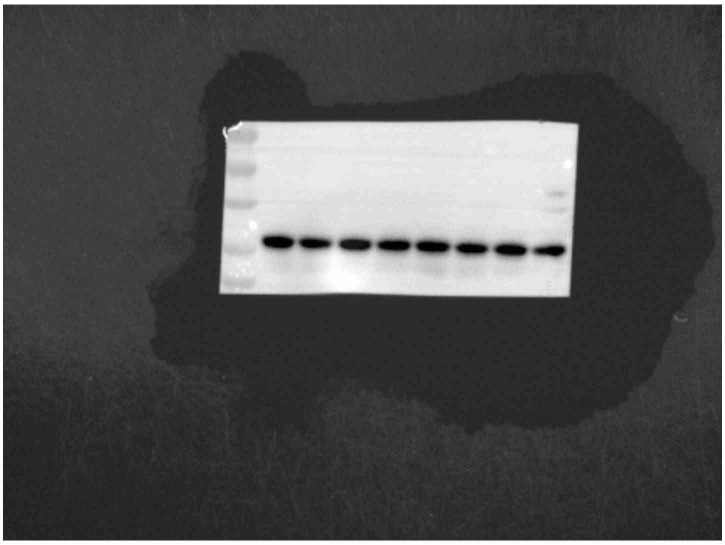

GAPDH

Figure6-H

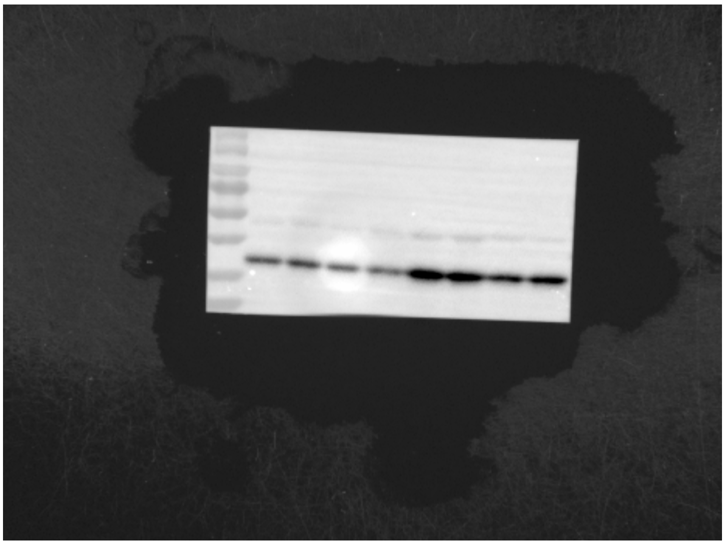

STING

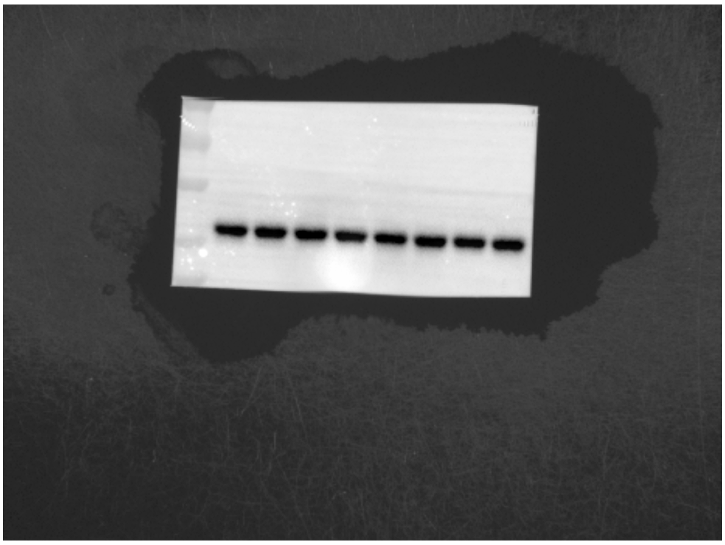

GAPDH

Figure7-A

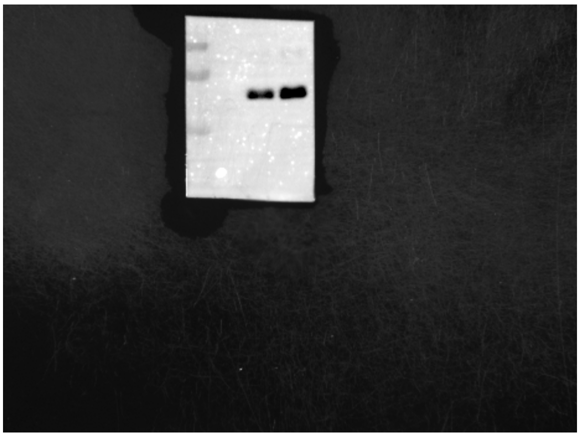

FLAG

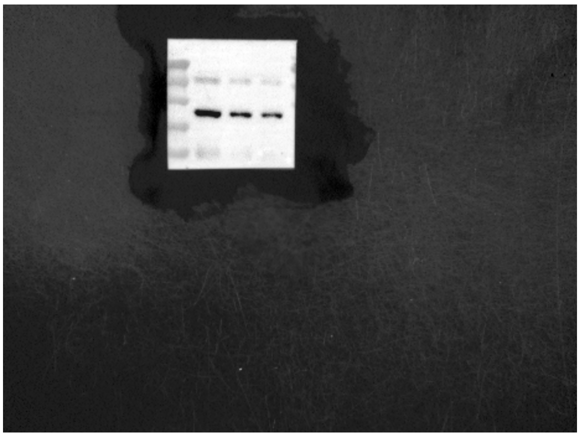

STING

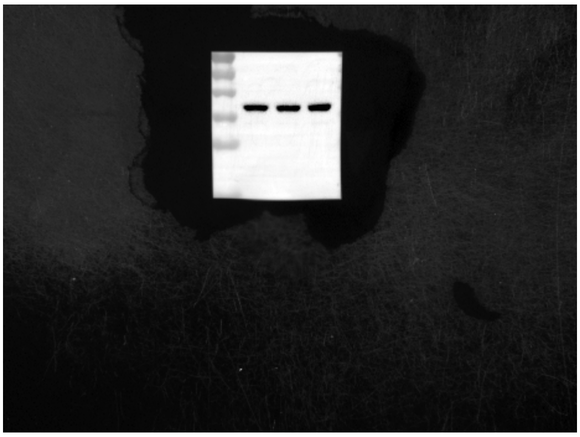

GAPDH

Figure7-B

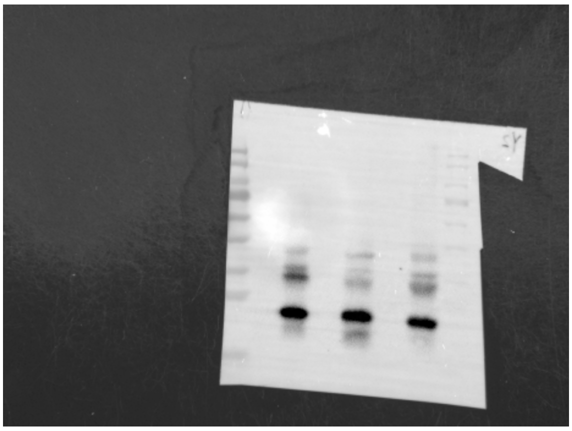

FLAG

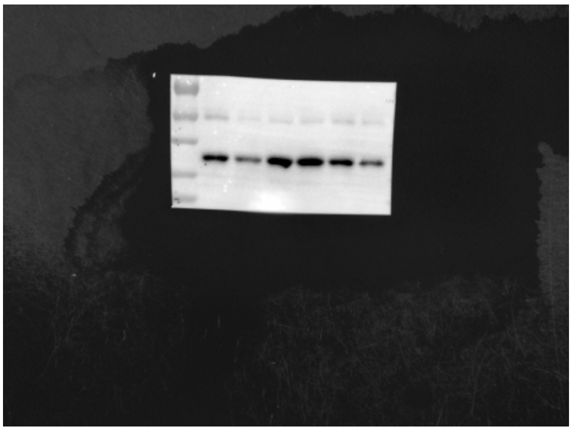

STING

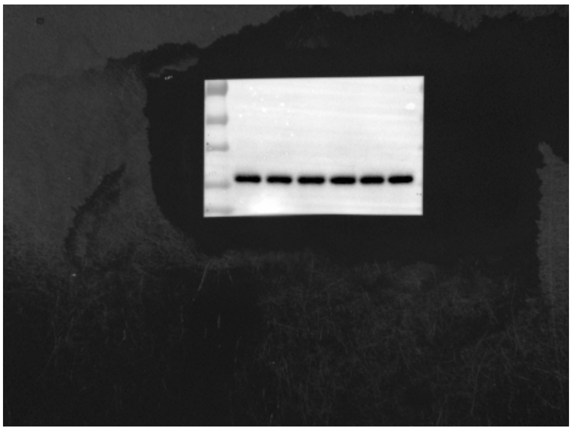

GAPDH

Figure7-C

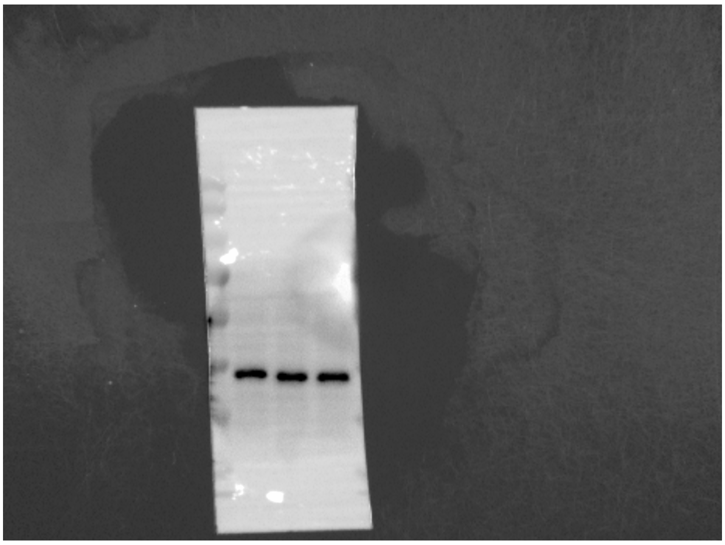

IN FLAG

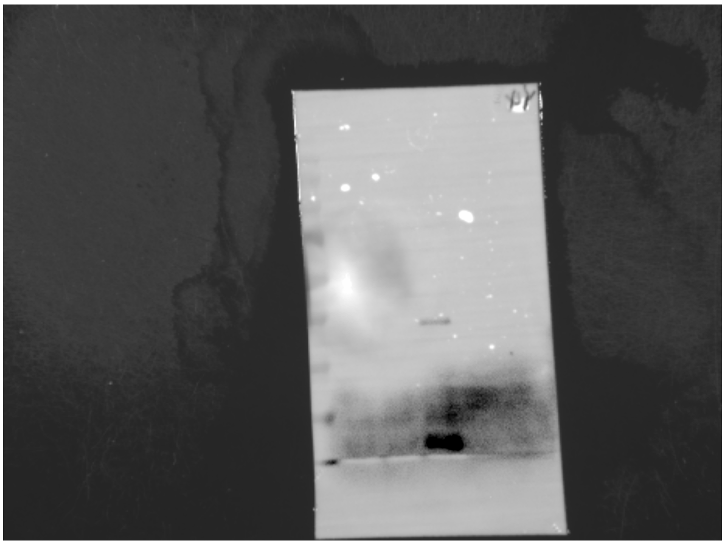

IN HA

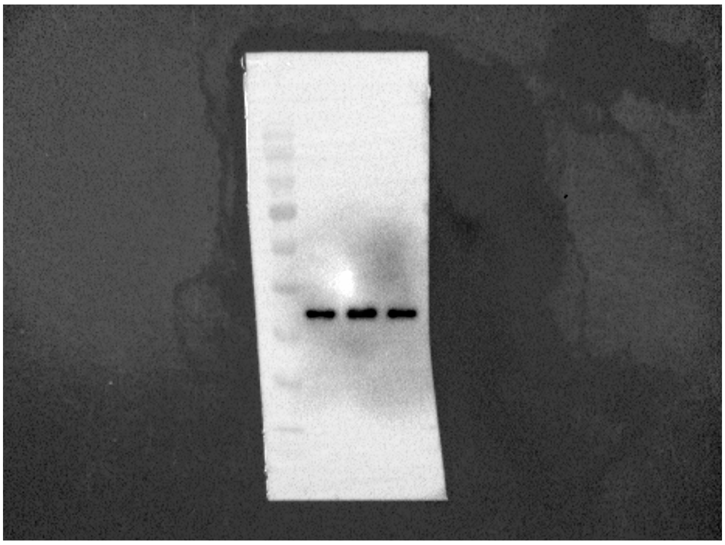

GAPDH

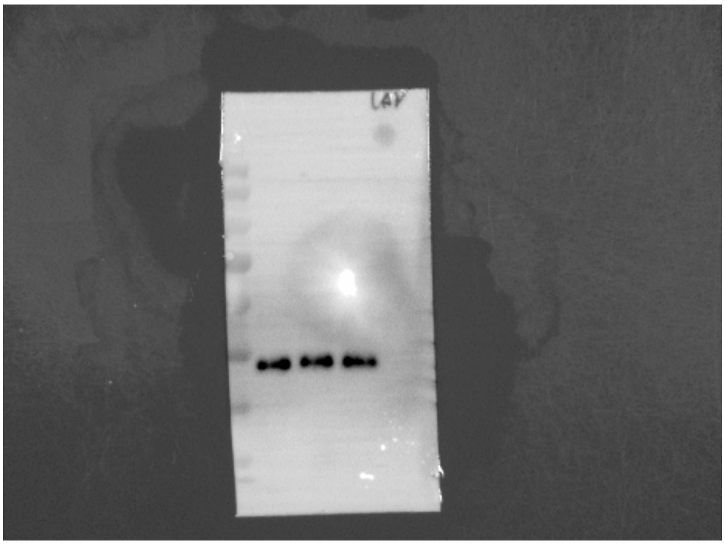

IP FLAG

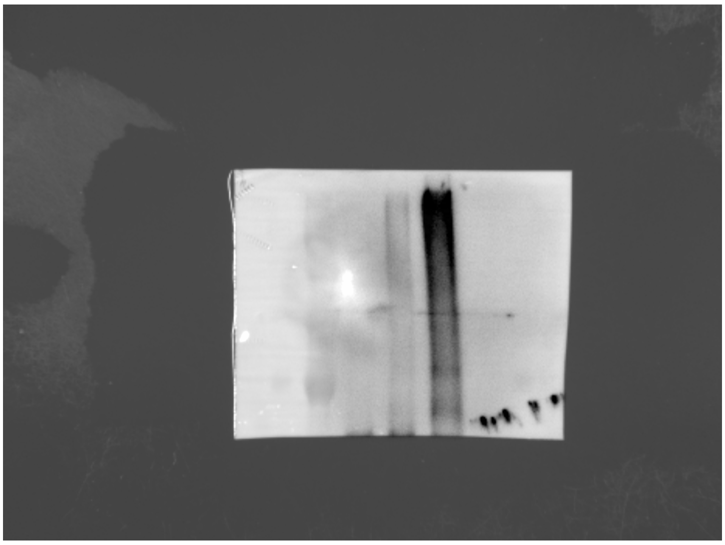

IP MYC

Figure7-D

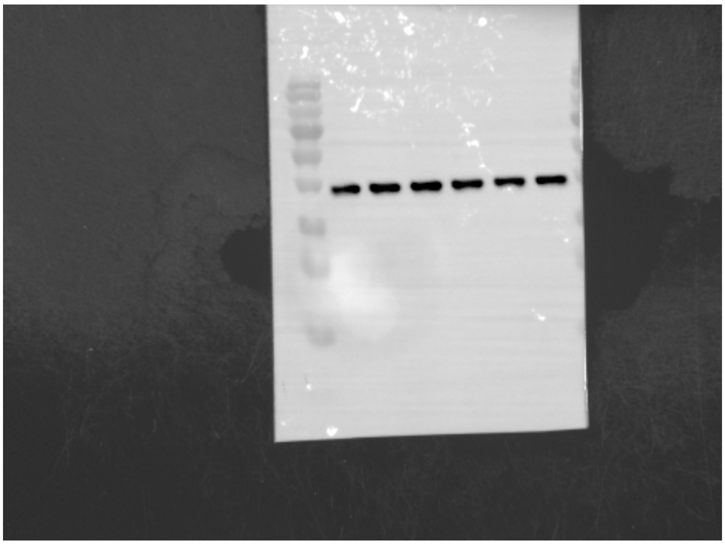

IN FLAG

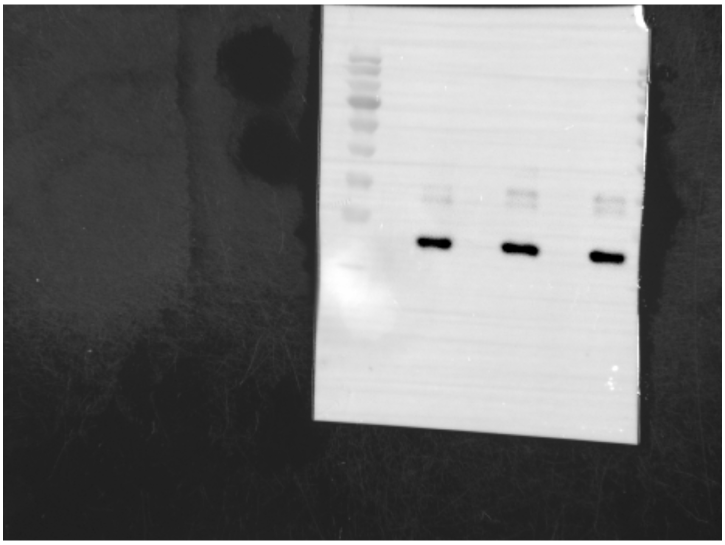

IN HA

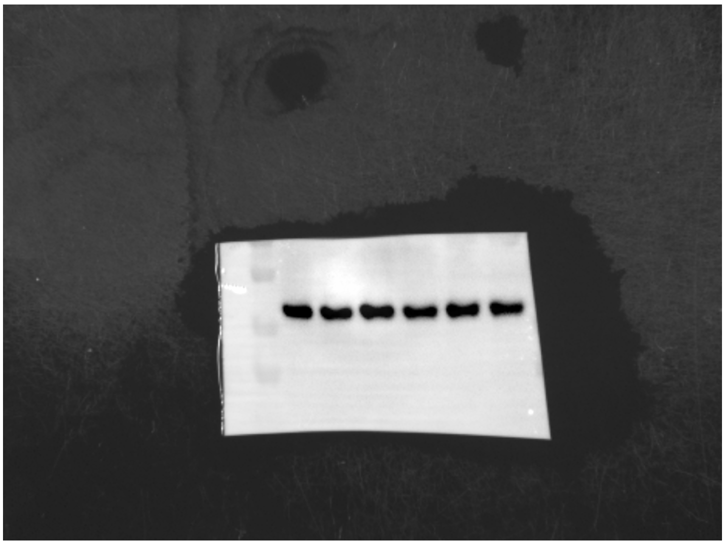

GAPDH

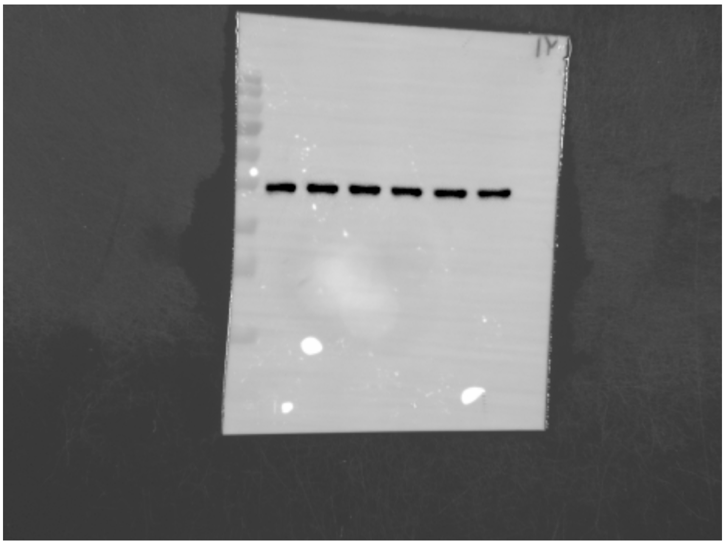

IP FLAG

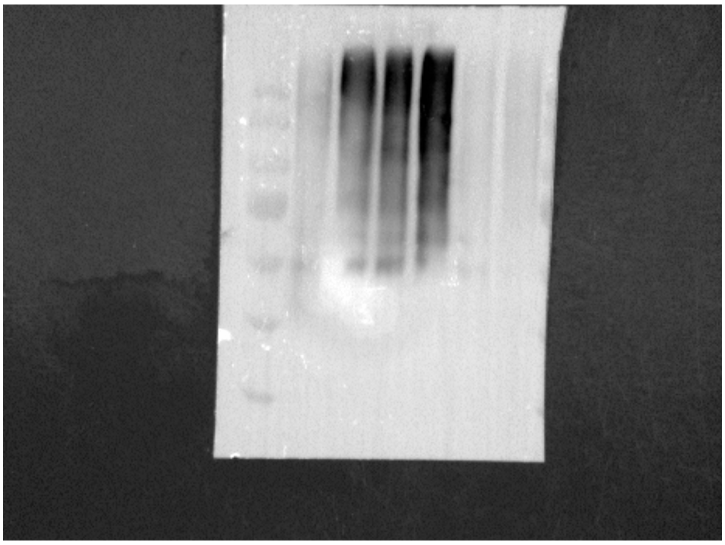

IP MYC

Figure7-E

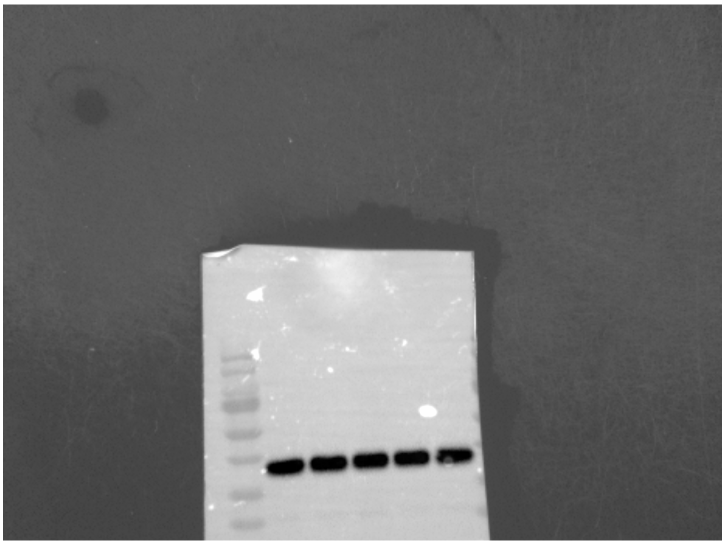

IN FLAG

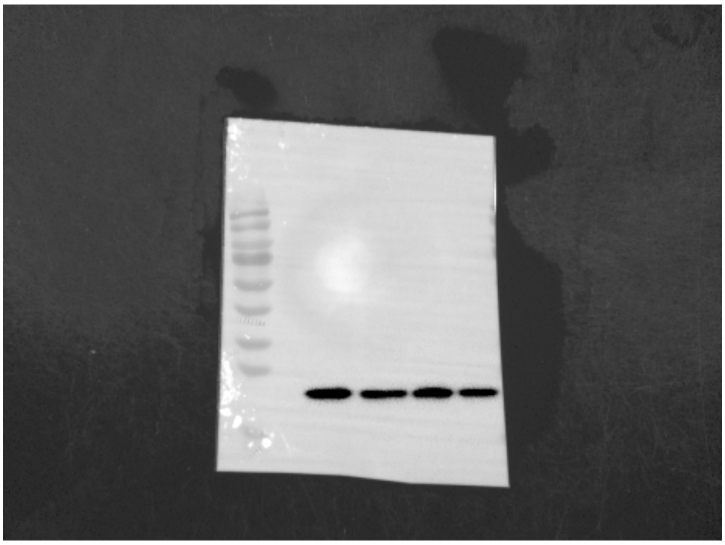

IN HA

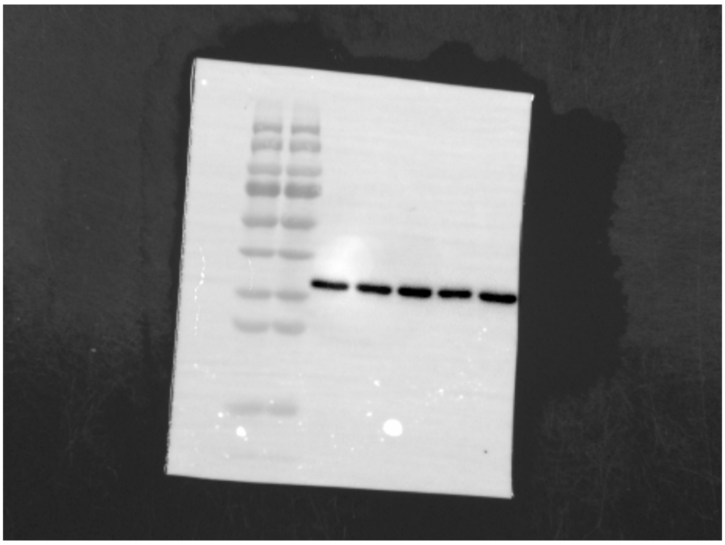

GAPDH

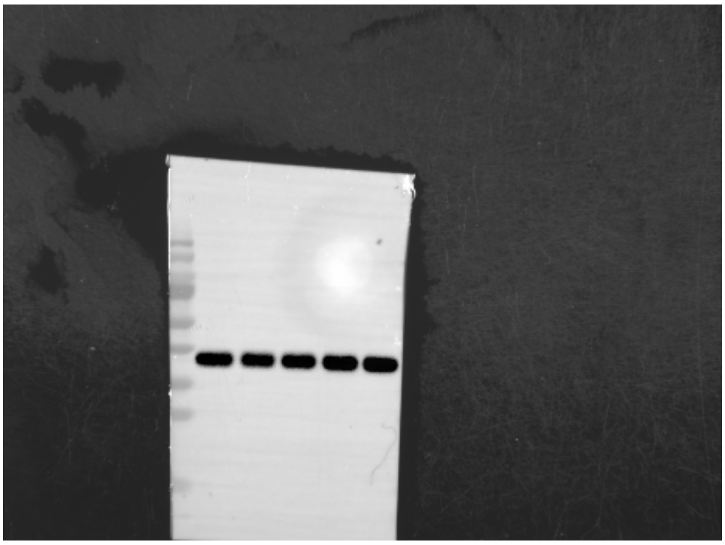

IP FLAG

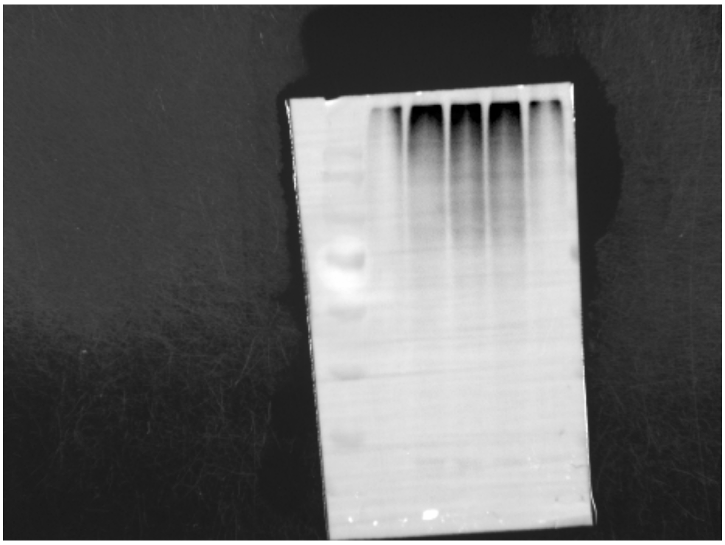

IP MYC

Figure7-F

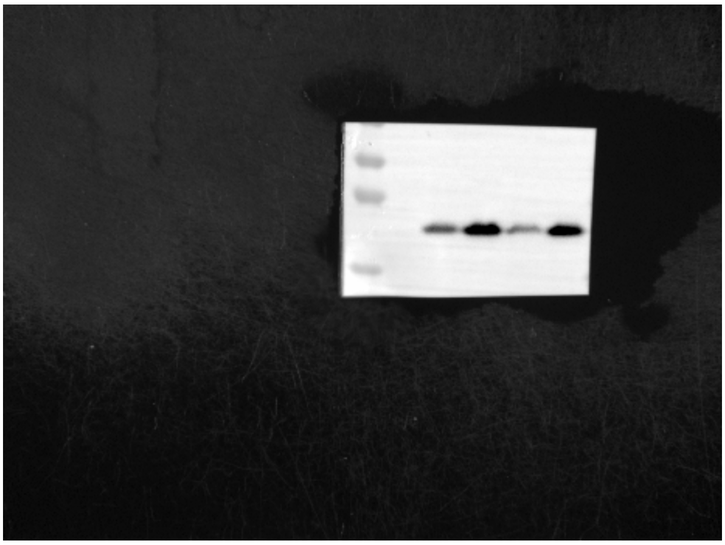

FLAG

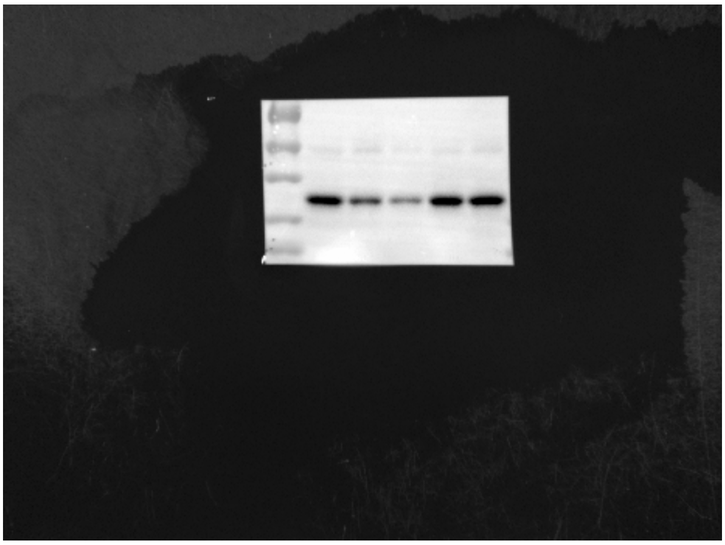

STING

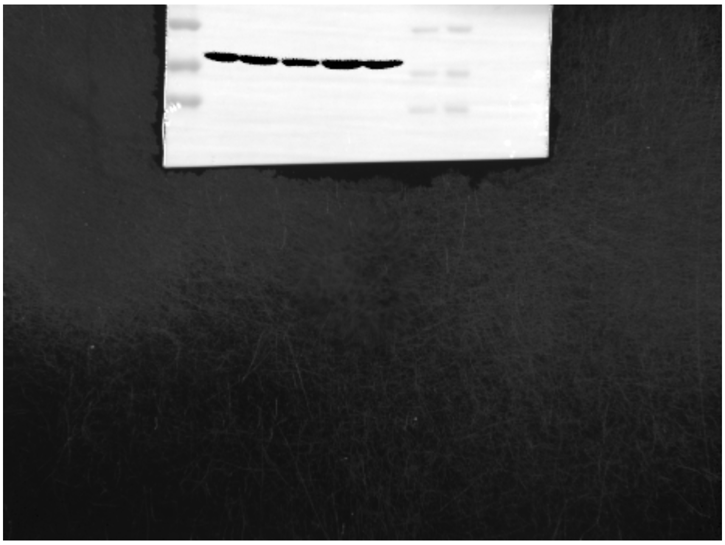

GAPDH

Figure7-G

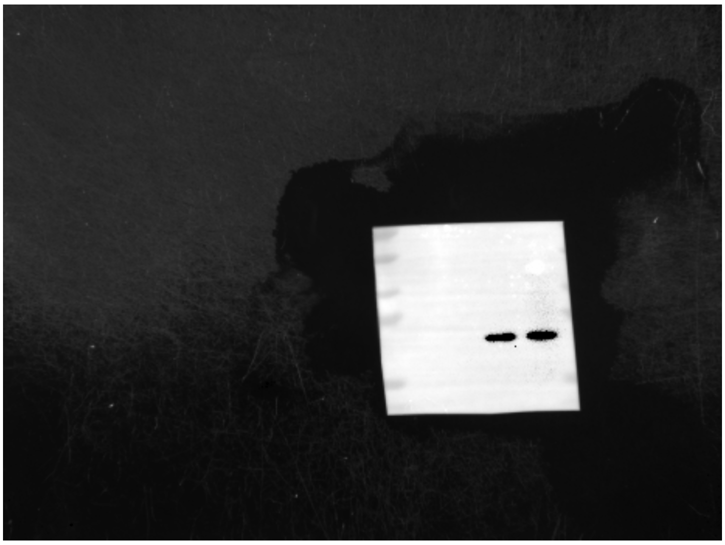

IN FLAG

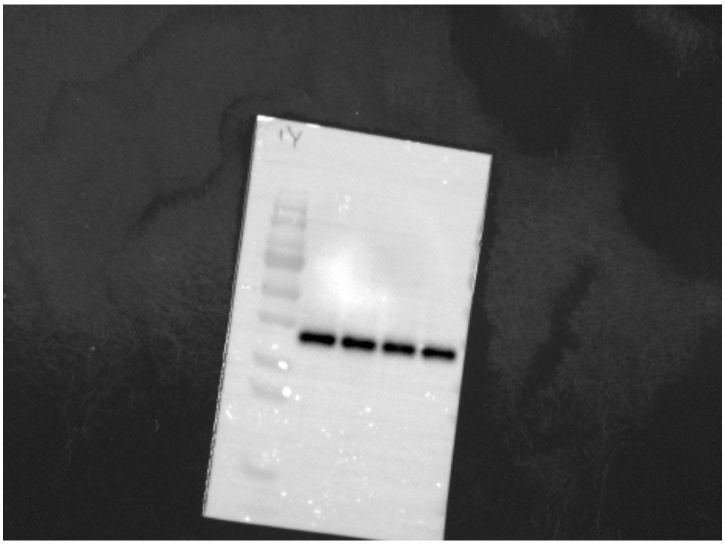

IN HA

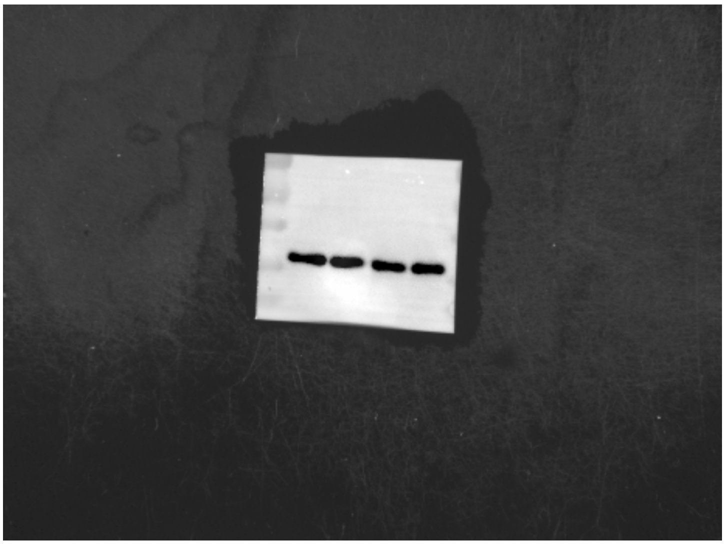

GAPDH

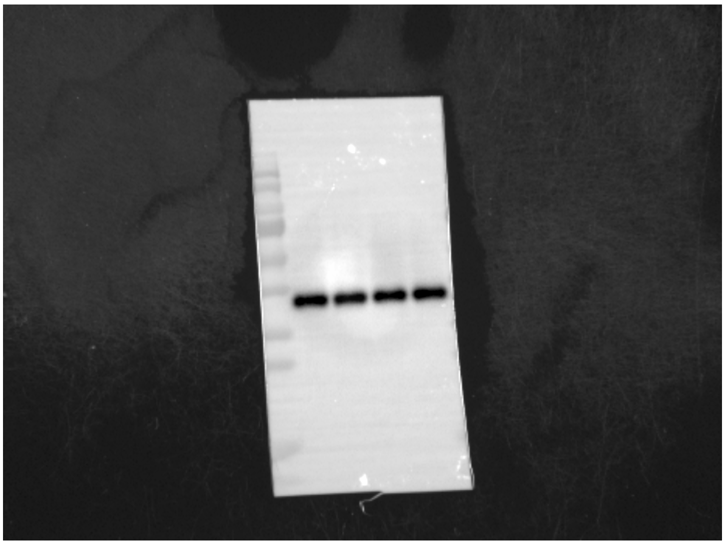

IP HA

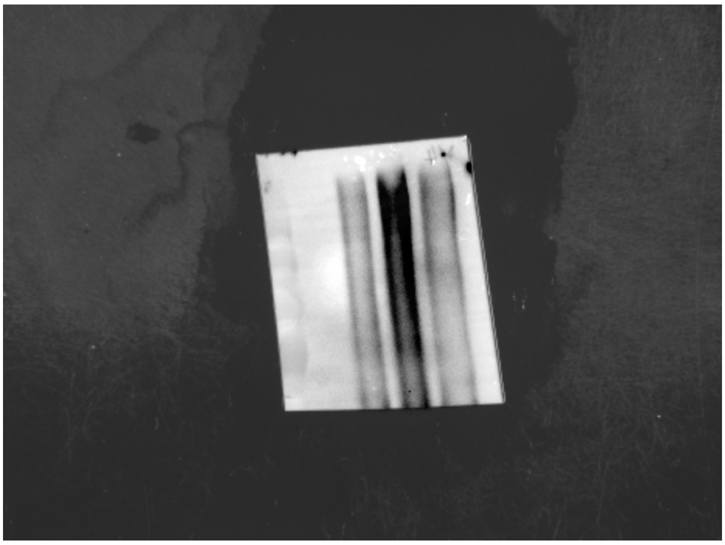

IP MYC

Figure7-I

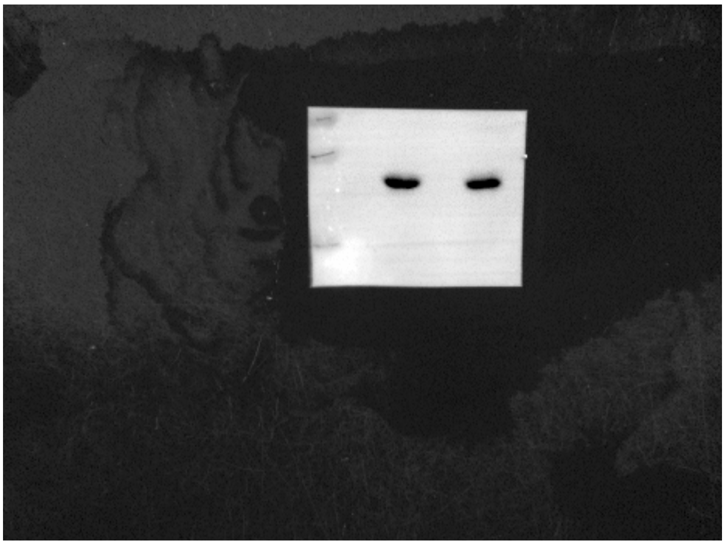

FLAG RNF5

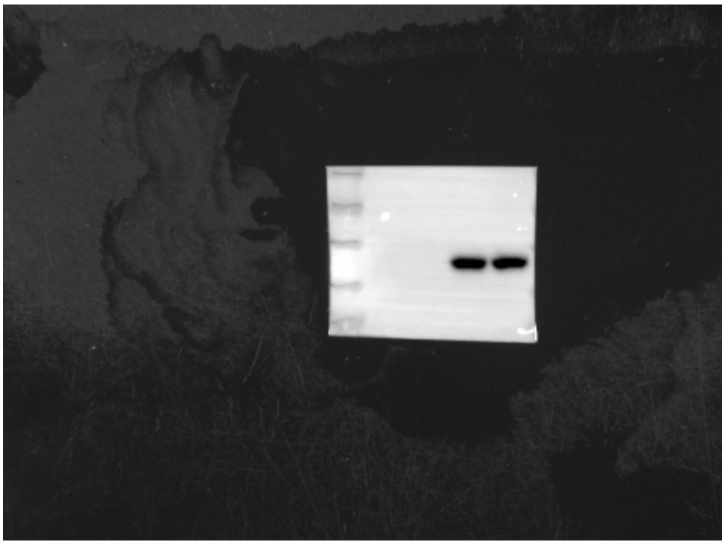

FLAG STING

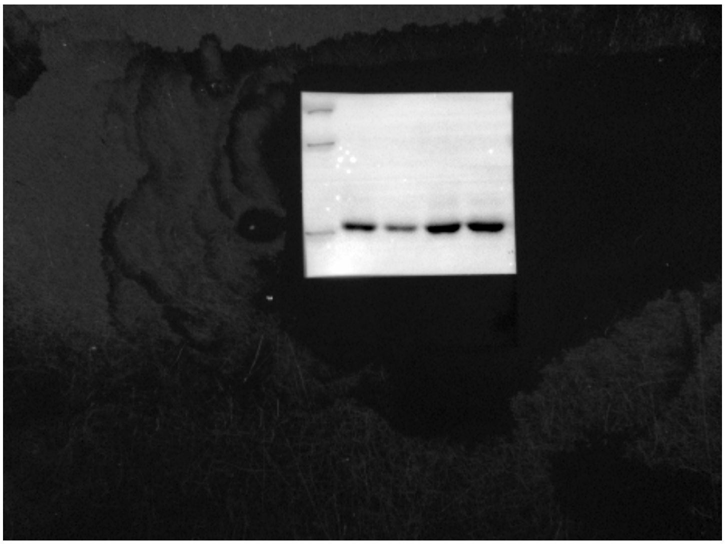

ANP

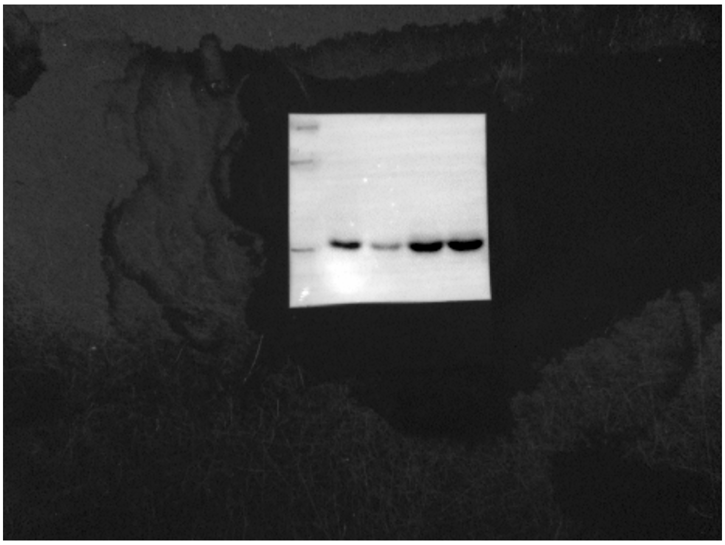

BNP

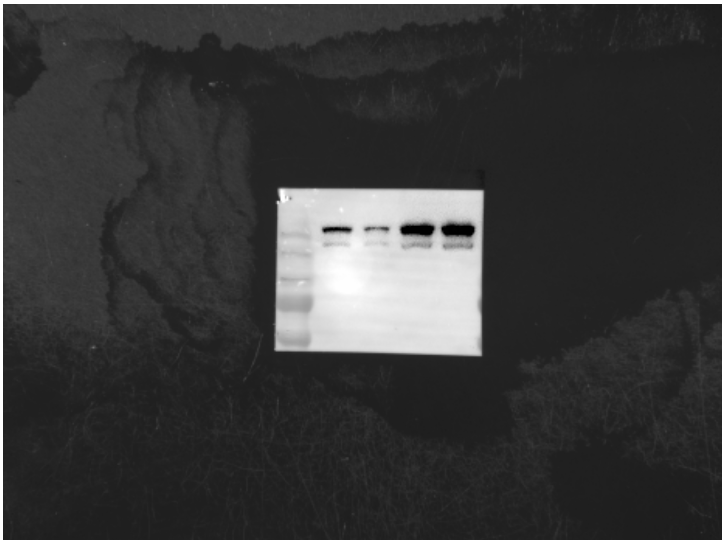

MYH7

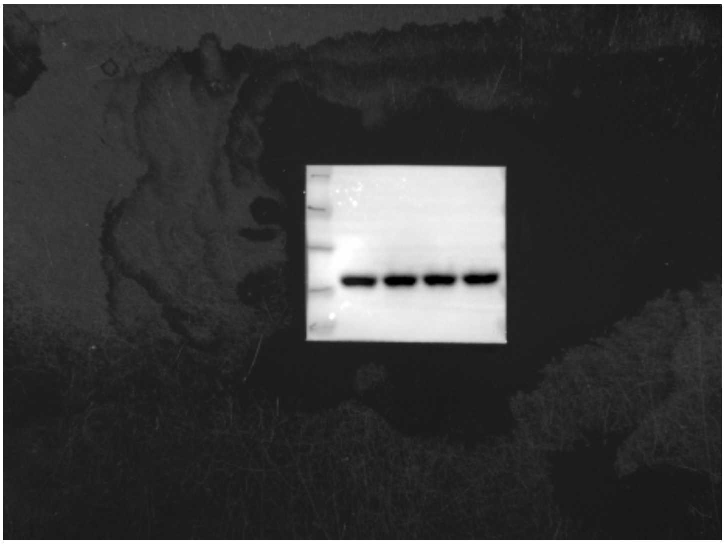

GAPDH
